# The Electrophilicity of Surface Carbon Species in the Redox Reactions of CuO‐CeO_2_ Catalysts

**DOI:** 10.1002/anie.202102570

**Published:** 2021-04-08

**Authors:** Liqun Kang, Bolun Wang, Andreas T. Güntner, Siyuan Xu, Xuhao Wan, Yiyun Liu, Sushila Marlow, Yifei Ren, Diego Gianolio, Chiu C. Tang, Vadim Murzin, Hiroyuki Asakura, Qian He, Shaoliang Guan, Juan J. Velasco‐Vélez, Sotiris E. Pratsinis, Yuzheng Guo, Feng Ryan Wang

**Affiliations:** ^1^ Department of Chemical Engineering University College London London WC1E 7JE UK; ^2^ Particle Technology Laboratory Institute of Process Engineering Department of Mechanical and Process Engineering ETH Zürich 8092 Zürich Switzerland; ^3^ Diamond Light Source Ltd Harwell Science and Innovation Campus Didcot Oxfordshire OX11 0DE UK; ^4^ Deutsches Elektronen Synchrotron DESY 22607 Hamburg Germany; ^5^ Department of Molecular Engineering Graduate School of Engineering Kyoto University Kyotodaigaku Katsura Nishikyo-ku Kyoto 6158510 Japan; ^6^ Department of Materials Science and Engineering National University of Singapore Singapore 117575 Singapore; ^7^ HarwellXPS—The EPSRC National Facility for Photoelectron Spectroscopy Research Complex at Harwell (RCaH) Didcot OX11 0FA UK; ^8^ Fritz-Haber-Institut der Max-Planck-Gesellschaft Faradayweg 4–6 14195 Berlin Germany; ^9^ School of Electrical Engineering and Automation Wuhan University Wuhan China

**Keywords:** CO oxidation, copper-ceria, electrophilicity, EMSI, surface chemistry

## Abstract

Electronic metal–support interactions (EMSI) describe the electron flow between metal sites and a metal oxide support. It is generally used to follow the mechanism of redox reactions. In this study of CuO‐CeO_2_ redox, an additional flow of electrons from metallic Cu to surface carbon species is observed via a combination of operando X‐ray absorption spectroscopy, synchrotron X‐ray powder diffraction, near ambient pressure near edge X‐ray absorption fine structure spectroscopy, and diffuse reflectance infrared Fourier transform spectroscopy. An electronic metal–support–carbon interaction (EMSCI) is proposed to explain the reaction pathway of CO oxidation. The EMSCI provides a complete picture of the mass and electron flow, which will help predict and improve the catalytic performance in the selective activation of CO_2_, carbonate, or carbonyl species in C1 chemistry.

## Graphical abstract

During the oxidation of CO over the surface of a CuO‐CeO_2_ catalyst, electrons are simultaneously transferred from Cu^0^ to lattice Ce^4+^ and surface carbon species deposited from CO. These “electronic metal–support–carbon interactions” (EMSCI) are thought to play an important role in the reactions involving the support and surface carbon species in C1 chemistry.

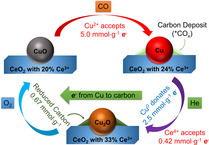

## Introduction

The interaction between metals and supports plays a vital role in modulating the catalytic performance of active sites. Concepts such as SMSI^[1]^ and EMSI^[2]^ are well‐established to describe the geometric,^[3]^ electronic[^2b^, ^2c^, ^2d^, ^4^] and bifunctional^[5]^ modification of active sites from the support. With respect to the electronic interaction, there is a flow of electrons either from a metallic centre to an oxidative support or from a reductive support to an oxidative metal centre. The net electron flow leads to modified electronic structures of the active centre and its surroundings, and thus to different behaviours in the adsorption and activation of reaction molecules compared to its unperturbed state. In addition to the oxide support, the destination of the electron flow can also be the surface carbon species resulting from the decomposition and deposition from carbonaceous reactants. Carbon atoms, with nine oxidation states and an electronegativity of 2.55, can accept electrons from metals. Carbon materials are widely used as electron acceptors in lithium‐ion batteries^[6]^ and organic photovoltaics.^[7]^ There are some representative studies in catalysis find electrons are transferred from metal to carbon,^[8]^ especially in electrocatalysis.^[9]^ Reduction of carbon can form metal‐carbon species in Fischer–Tropsch synthesis^[10]^ and alkynes hydrogenation.^[11]^ A CO_2_^δ−^ species is formed when the 2π_u_ orbital of surface CO_2_ accepts electrons from metallic Cu.^[12]^ Theoretical calculations also predict the modification of the Cu electronic structure via surface carbons.^[13]^

Cu has moderate adsorption strength for carbonaceous intermediates,^[14]^ and is widely used for C1 chemistry, including CO oxidation,^[15]^ water‐gas shift,^[16]^ steam reforming,^[17]^ methanol synthesis,^[18]^ and electrochemical reduction of CO_2_.[^14^, ^19^] The well‐documented Ce^4+^/Ce^3+^ redox pair enables the transfer of oxygen atoms and electrons between Cu^2+^/Cu^+^/Cu^0^ active sites and the CeO_2_ support.^[20]^ Validation of this catalytic cycle still requires precise quantification of their oxidation states to match the balance of electron transfer.^[21]^ Here we report the full picture of electron transfer in this system by considering the electrophilicity of surface carbon species, which are in situ deposited from CO molecules. At 453 K, electrons are initially enriched on metallic Cu via CO reduction (*CO stage*) to build up the chemical potential for an electron flow within the catalyst. The electrophilicity of surface carbon species is then studied in an inert atmosphere (*He stage*) to exclude electron transfer from or to the gaseous molecules. Finally, O_2_ is used to extract electrons that are originally injected into the catalyst from CO (*O_2_ stage*). The CO oxidation activity of the catalyst at individual stage is compared below 353 K to elucidate the impact of initial oxidation states of Cu and Ce as well as the surface carbon species.

**Figure 1:**
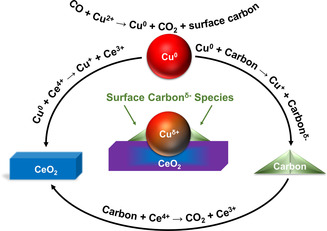
Schematic of the electronic metal–support–carbon interactions (EMSCI) among Cu^0^, CeO_2_, and surface carbon species. The models are simplified to illustrate the major structural changes of the sample. Surface carbon species are represented by the shaded green triangles. They are located on the surface of Cu and CeO_2_, as well as at the interface.

In a full cycle of CO oxidation, electrons are transferred from CO to Cu, then to Ce and carbon (Figure 1) and finally to O_2_. We therefore extend the concept of EMSI to “electronic metal‐support‐carbon interactions” (EMSCI) in order to address this interplay among Cu, Ce and carbon species.

## Results and Discussion

### Highly Dispersed CuO Clusters on CeO_2_

Fine powder of highly dispersed 20 wt % CuO clusters on CeO_2_ with 71 m^2^ g^−1^ specific surface area were prepared via flame spray pyrolysis.[^20b^, ^22^] The high‐resolution aberration‐corrected high angle annular dark field‐scanning transmission electron microscopy (HAADF‐STEM) images show crystalline CeO_2_ particles with an average size of around 6 nm (Figure S1f,g). The corresponding energy‐dispersive X‐ray spectroscopy (EDS) maps identify the presence of Cu in the form of ca. 5 nm particles surrounded by CeO_2_ (Figure S1a‐e). The X‐ray photoelectron spectroscopy (XPS) shows that ca. 18 % of the Ce is Ce^3+^, indicating a considerable number of oxygen vacancies in the lattice (Figure S2 and Table S1).^[23]^ The surface Cu/(Cu+Ce) ratio determined by XPS is 0.36, which is close to the theoretical bulk Cu/(Cu+Ce) ratio (0.35) for 20 wt % CuO‐CeO_2_. This indicates good dispersion of the Cu species over CeO_2_, leading to large Cu/Ce interface. In comparison, Cu species are usually enriched on CeO_2_ surface using conventional synthesis methods, showing much higher surface Cu content compared to the bulk composition (Table S2).^[24]^ Laboratory X‐ray diffraction (XRD) pattern shows broadened CeO_2_ diffraction peaks (Figure S3a), whereas weak CuO diffraction peaks can be recognised by synchrotron X‐ray powder diffraction (SXPD; Figure S3b). Therefore, the initial catalyst structure of 5 nm CuO and 6 nm CeO_2_ is confirmed with 18 % Ce^3+^ present.

This 20 wt % CuO‐CeO_2_ catalyst has been applied for the preferential CO oxidation in the presence of 50 % H_2_, achieving a wide temperature window from 377 to 388 K with 99 % conversion and selectivity of CO oxidation.^[20b]^ Our previous in situ study used a sequence of CO/N_2_/O_2_ flows at 453 K to probe the change of Cu oxidation state and the corresponding gas profile.^[20b]^ Two interesting phenomena were observed: 1) under inert N_2_, Cu^0^ was slowly reoxidised; 2) when N_2_ was changed to O_2_, CO_2_ was released without an external CO feed. In addition, the near ambient pressure‐near‐edge X‐ray absorption fine structure (NAP‐NEXAFS) spectra of a similar system reveals the conversion from oxidised carbon species (288.3 eV) to reduced carbon species (284.9 eV) in ultra‐high vacuum (Figure S4). We hypothesise that carbon species will deposit on the surface of CuO‐CeO_2_ from gas‐phase CO. Such carbon species are reduced under inert conditions, whereas Cu^0^ is oxidised simultaneously. When O_2_ is introduced, the carbon species are oxidised to CO_2_. This hypothesis of EMSCI is verified via a combination of operando X‐ray absorption fine structure (XAFS), SXPD, NAP‐NEXAFS, diffuse reflectance infrared Fourier transform spectroscopy (DRIFTS) and gas component quantification at the CO, He and O_2_ stages, as presented below.

### CO Stage: Electron Transfer from CO to Cu

**Figure 2:**
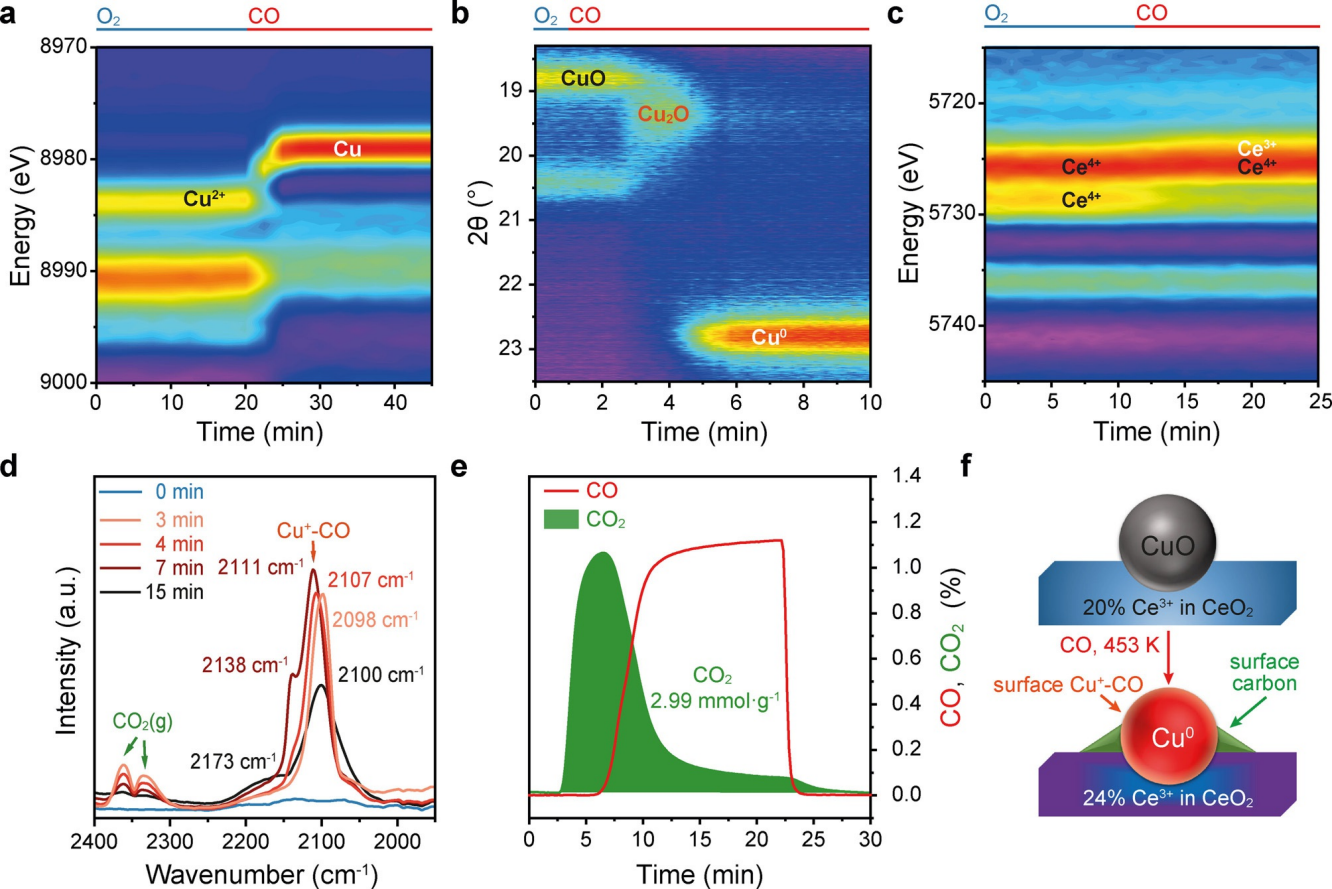
Operando characterisation of Cu, Ce and surface carbon during the reduction of CO by CuO‐CeO_2_ at 453 K. a) Contour map of the Cu K‐edge first derivative XANES spectra, showing the reduction of Cu^2+^ to Cu^0^. XANES spectra are shown in Figure S5. b) Contour map of the SXPD patterns, showing the conversion of CuO to metallic Cu. The SXPD patterns are shown in Figure S7. c) Contour map of the Ce L_3_‐edge first derivative XANES spectra, showing the increase of Ce^3+^ content after reduction. XANES spectra are shown in Figure S8 and their fitting results are given in Table S3. d) DRIFTS spectra of the surface carbonyl species from CO adsorption. The initial spectrum obtained in O_2_ at 453 K is labelled as 0 min (blue curve) and the progress in CO at 453 K is colour coded from light pink to black. e) Exhaust gas profile of the CO reduction. The 80 mg sample was pre‐oxidised in 20 % O_2_ then purged with N_2_ at 453 K. f) Simplified models of the structural evolution from CuO‐CeO_2_ to Cu^0^‐CeO_2_.

Introducing CO at 453 K to the catalyst reduces Cu^2+^ and Ce^4+^ (Figure 2). Most of the Cu reduction follows the sequence of Cu^2+^→Cu^+^→Cu^0^, as indicated in the Cu K‐edge X‐ray absorption near edge structure (XANES) and SXPD (Figure 2 a,b). The Ce^3+^ content increases from 20 % to 24 % according to the peak fitting results of the Ce L_3_‐edge XANES (Figure 2 c, note that the initial 20 % Ce^3+^ content is slightly different from the 18 % Ce^3+^ obtained from XPS study).

DRIFTS shows the absorption peaks of Cu^+^‐carbonyl species (Cu^+^‐CO) in the range of 2160‐2080 cm^−1^,^[25]^ suggesting the presence of Cu^+^ on the surface of metallic Cu (Figure 2 d). NAP‐NEXAFS at the Cu L_3_‐edge validates that Cu^+^ dominates the surface layer after the reduction of CuO (Figure S10). Such surface Cu^+^ species on Cu^0^ clusters over CeO_2_ have also been reported in the water gas shift reaction.^[16]^ In general, the surface CO absorption band undergoes a slight blue shift from 2098 to 2111 cm^−1^, and then a red shift to 2100 cm^−1^ with a significant intensity decrease. The initial blue shift indicates the increase of CO coverage^[25]^ on Cu^+^ along with the release of CO_2_ detected at 2364 and 2337 cm^−1^. A new shoulder peak at 2138 cm^−1^ appears when the CO coverage is maximised. The peak is similar to the reported band at 2135 cm^−1^ of CO on partially reduced CuO_x_.^[25b]^ The increase of a broad feature between 1700‐1200 cm^−1^ (Figure S11 from blue to dark red) that corresponds to surface carbonate species is observed.[^16b^, ^26^] The formation of the carbonate stems from the oxidation of surface carbonyl species, a process that is reported in the literature.^[16b]^ These carbonates can increase the electrophilicity of neighbour Cu^+^,^[25a]^ leading to a broad absorption band of Cu^+^‐CO at 2173 cm^−1^ when the reduction of Cu is complete as reflected by the disappearance of CO_2_ peaks (Figure 2 d). The second stage red‐shift to the steady and broadened band centred at 2100 cm^−1^ corresponds to a combination of Cu^+^‐CO and Cu^0^‐CO bands at 2160‐2080 cm^−1^ and 2090‐2060 cm^−1^, respectively.^[25]^ A slight decline in surface carbonate species is observed (Figure S11 from dark red to black).

The CO reduction of 20 wt % CuO‐CeO_2_ releases 2.99 mmol g^−1^ of CO_2_ at 453 K (Figure 2 e). Theoretically, the complete reduction from Cu^2+^ to Cu^0^ will generate 2.52 mmol g^−1^ of CO_2_. Furthermore, there is a 4 % reduction of Ce^4+^ to Ce^3+^ (according to XANES fitting) results in 0.09 mmol g^−1^ of CO_2_ formation. These results indicate an additional 0.38 mmol g^−1^ CO_2_ generation that is not related to the Cu and Ce redox. According to the literature, this additional CO_2_ may come from the disproportionation of CO.^[27]^ A very recent work on CO_2_ methanation also finds that the presence of Ce^3+^ can also help dissociate CO and CO_2_, leading to diverse carbon species.^[28]^ Carbonyls and carbonates are detected by DRIFTS, suggesting the high complexity of those surface carbon species. (Figure 2 d and Figure S11).

The combination of operando XAFS of the Cu and Ce oxidation states, SXPD of small crystalline clusters and DRIFTS of surface carbon species reveals the change of Cu, Ce and C during the *CO stage* (Figure 2 f). The majority of CuO is fast reduced to metallic Cu with a trace amount of surface Cu^+^, whereas 4 % Ce^4+^ is reduced to Ce^3+^. Surface carbon species (i.e., carbonyls and carbonates) are deposited from gaseous CO.

### He Stage: Electron Transfer from Cu^0^ to Ce^4+^ and Surface Carbon

**Figure 3:**
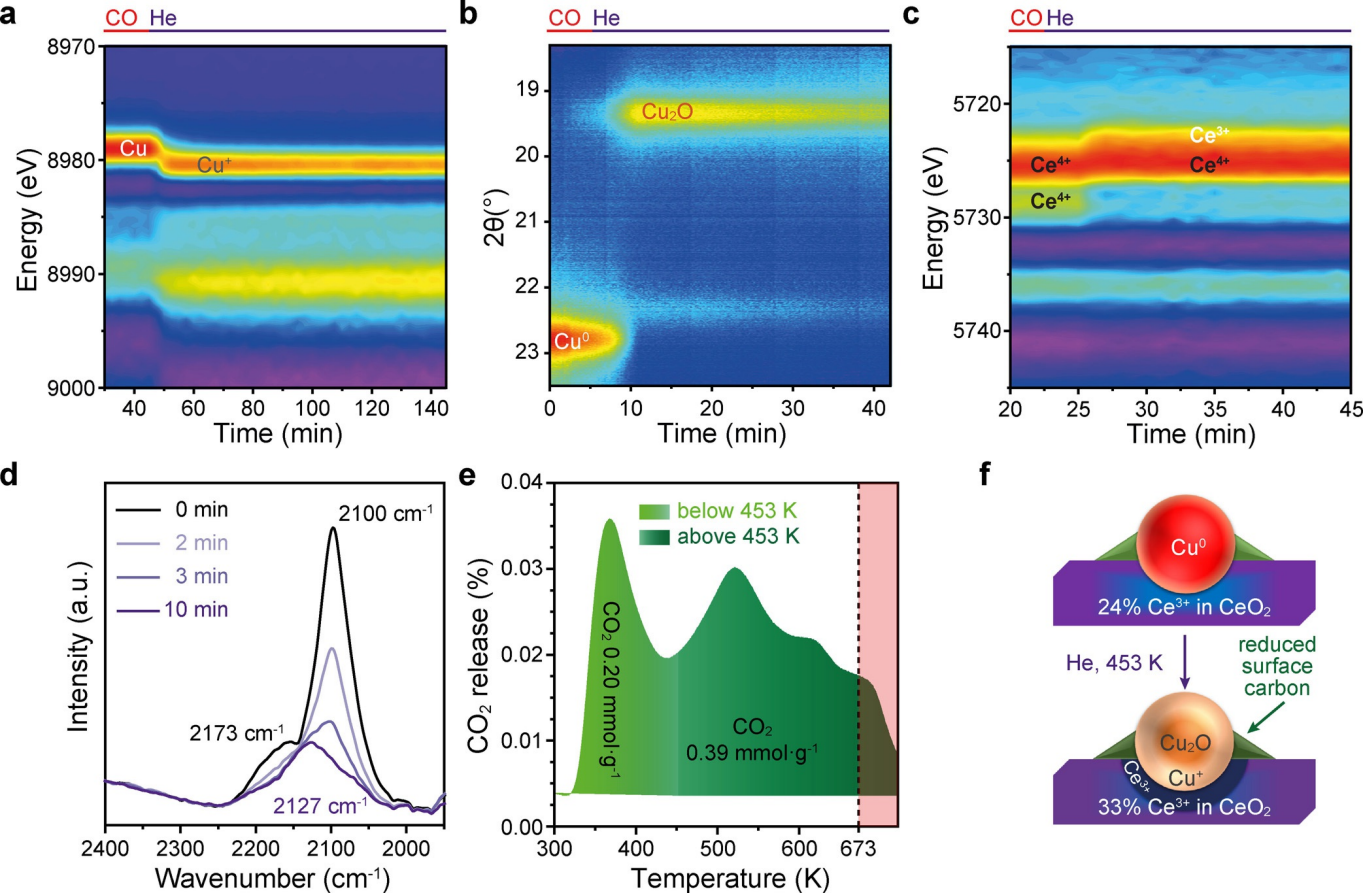
Operando characterisation of Cu, Ce and surface carbon during the internal reaction of Cu^0^‐CeO_2_ at 453 K in He. a) Contour map of the Cu K‐edge first derivative XANES spectra, showing the oxidation of Cu^0^ to Cu^+^. XANES spectra are shown in Figure S12. b) Contour map of the SXPD patterns, showing the conversion of metallic Cu to Cu_2_O and the following amorphization of Cu_2_O. SXPD patterns are shown in Figure S13. c) Contour map of the Ce L_3_‐edge first derivative XANES spectra, showing the further increase of Ce^3+^ content by accepting electrons from Cu^0^. XANES spectra are shown in Figure S14 and their fitting results are given in Table S3. d) DRIFTS spectra of the surface carbonyl species after CO desorption. The initial spectrum obtained in CO at 453 K is labelled as 0 min (black curve) and the progress in He at 453 K is colour coded from light violet to purple. e) Exhaust gas profile during TPD from 300 K to 673 K, then holding at 673 K for 30 min indicated by the pink‐shaded region. The CO released during TPD is negligible. f) Simplified models of the structural evolution from Cu^0^‐CeO_2_ to Cu^+^‐CeO_2_.

The inert He flow enables observation of the internal reaction among Cu^0^, Ce^3+^/Ce^4+^ and surface carbon species. Cu^0^ clusters are immediately oxidised to Cu^+^ once CO is replaced by He (Figure 3 a,b). Simultaneously, 9 % Ce^4+^ is reduced to Ce^3+^ (Figure 3 c). These results suggest a fast redox reaction [Eq. 1].

(1) $$\mathrm{Cu}{}^{0}+\mathrm{Ce}{}^{4+}\to \mathrm{Cu}{}^{+}+\mathrm{Ce}{}^{3+}$$

To the best of our knowledge, the observation of Cu oxidation and Ce reduction here is the first direct evidence for this reaction, proving the EMSI between Cu and Ce. The Cu^+^ state is stable in the He flow (Figure 3 a), whereas the crystalline Cu_2_O feature gradually decreases with time at 453 K, suggesting the amorphization of Cu_2_O (Figure 3 b). The oxidation of all Cu^0^ to Cu^+^ would require 54 % Ce^4+^ to be reduced to Ce^3+^, which is much higher than the 9 % formation of Ce^3+^. Within the system, the only destination to receive the additional electrons from Cu^0^ are surface carbon species with their abundant valence states, given that both carbonyl species and carbonate species are present on Cu^+^‐CeO_2_ at the *He stage* (Figure 3 d, Figure S15). The blue shift from 2100 cm^−1^ to 2127 cm^−1^ suggests the increased electrophilicity of Cu due to its oxidation. The surface carbon species as electron acceptors have also been reported for CO_2_ activation on metallic Cu, where CO_2_^δ−^ species were formed via electron transfer from Cu^0^ into the 2π_u_ orbital of surface CO_2_.^[12]^ Such CO_2_^δ−^ species were identified by surface enhanced Raman spectroscopy^[29]^ and XPS.^[30]^ In addition, the decomposition of these carbon species can generate surface O atoms to form Cu−O bond.[^26^, ^31^]

The flow of electrons from Cu^0^ to Ce^4+^ and surface carbon species under inert atmosphere proves the EMSCI concept (Figure 3 f). Temperature programmed desorption (TPD) is applied to support the presence of those surface carbon species at 453 K in addition to the DRIFTS evidence. To obtain a similar metallic Cu and CeO_2_ surface, the catalyst is reduced first with CO at 453 K and then cooled to 298 K under the CO atmosphere. Subsequent heating in He leads to CO_2_ desorption. The released CO_2_ may come from the decomposition of surface carbonates^[26]^ or the desorption of CO_2_ formed during CO reduction.^[31]^ 0.20 mmol g^−1^ and 0.39 mmol g^−1^ of CO_2_ release are observed below and above 453 K, respectively (Figure 3 e). The latter is stable under He in the in situ study and is partially responsible for the oxidation of Cu (Figure 3 a,b). The remaining surface carbon species that cannot be desorbed by heating in He may also accept electrons and oxidise Cu. Density functional theory (DFT) calculations with Bader charge analysis are performed to evaluate the electron transfer between Cu^0^ and adsorbed carbon species. Each Cu atom that adsorbs CO has extra +0.11e and +0.12e at low and high CO coverages, respectively whereas each CO molecule gains −0.13e from Cu (Figure S16, S17 and Table S4). When the coverage of carbon species is further increased, the adsorbed carbon atoms, the number of which equals to half of the Cu atoms, gain −16.58e from Cu, resulting in +0.20e to +0.70e on individual Cu atom (Figure S18). The theoretical calculations results validate the electrophilicity of surface carbon species and its impact on reducing the electron density of Cu. An *O_2_ stage* is then carried out to study those residual carbon and the further oxidation of Cu^+^/Ce^3+^.

### O_2_ Stage: Electron Transfer from Ce^4+^, Surface Carbon, and Cu^+^ to O_2_

**Figure 4:**
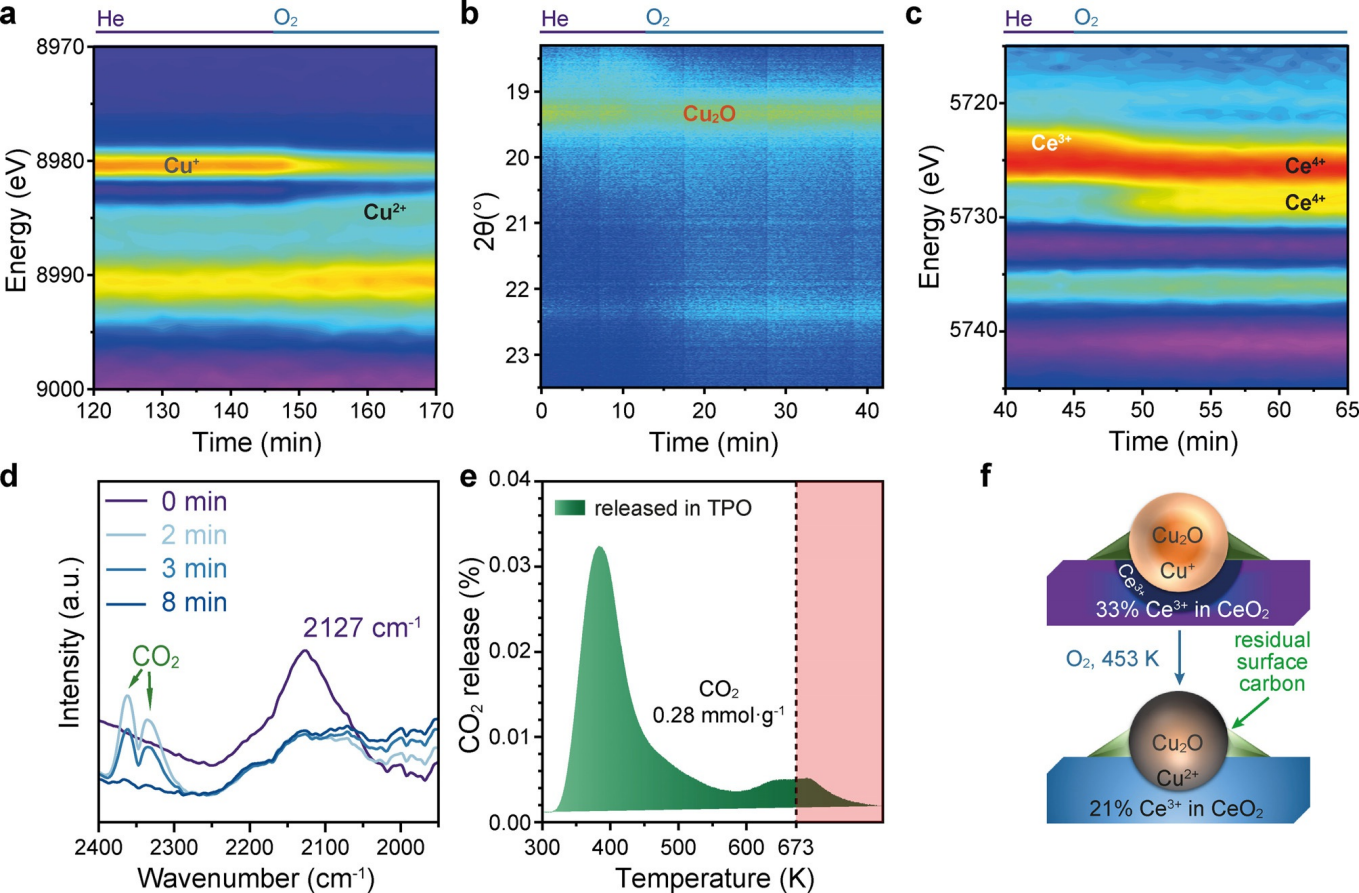
Operando characterisation of Cu, Ce and surface carbon during the oxidation of Cu^+^‐CeO_2_ at 453 K with O_2_. a) Contour map of the Cu K‐edge first derivative XANES spectra, showing the incomplete oxidation of Cu^+^ to Cu^2+^. XANES spectra are shown in Figure S19. b) Contour map of the SXPD patterns, showing the crystalline Cu_2_O preserved during oxidation. SXPD patterns are shown in Figure S20. c) Contour map of the Ce L_3_ edge first derivative XANES spectra, showing the recovery of Ce^3+^ content after oxidation. XANES spectra are shown in Figure S21 and their fitting results are given in Table S3. d) DRIFTS spectra of the surface carbonyl species as they are oxidised and released as CO_2_. The initial spectrum obtained in He at 453 K is labelled in purple and the progress in O_2_ at 453 K is colour coded from light blue to blue. e) Exhaust gas profile shows the released CO_2_ during TPO from 300 K to 673 K, then holding at 673 K for 30 min indicated by the pink‐shaded region. f) Simplified models of the structural evolution from Cu^+^‐CeO_2_ to Cu^+^/Cu^2+^‐CeO_2_.

When switching to O_2_ at 453 K, the Ce^3+^ content recovers to the initial level, whereas only a slight increase of Cu^2+^ is found (Figure 4 a,c). No significant change is found in SXPD, suggesting Cu^+^ remains amorphous (Figure 4 b). While the majority of Cu remains in Cu^+^ state, a full reoxidation to Cu^2+^ is found on the surface as indicated in the NAP‐NEXAFS (Figure S22). A possible explanation is that a dense layer of CuO is formed over the Cu_2_O surface, preventing further oxidation of Cu^+^. We hypothesise that when the size of the Cu_2_O decreases, the curvature of the surface CuO layer increases and more Cu can be oxidised into Cu^2+^. An extreme case is the atomic Cu sites, which can be completely oxidised back to Cu^2+^ even under He flow.^[22]^

The residual surface carbonyl species with a band at 2137 cm^−1^ are removed via oxidation with simultaneously released CO_2_ (Figure 4 d,f), whereas the carbonates only slightly decrease under O_2_ at 453 K (Figure S23). In addition to the 0.59 mmol g^−1^ CO_2_ released during TPD in He up to 673 K (Figure 3 e), temperature programmed oxidation (TPO) of the residual surface carbon can further generate 0.28 mmol g^−1^ CO_2_ (Figure 4 e). Therefore, at least 0.87 mmol g^−1^ of carbon species can be deposited and 0.67 mmol g^−1^ of them can contribute to the oxidation of Cu^0^ via EMSCI. The amount of carbon deposited on the surface may be directly proportional to the CO pressure (*P*_CO_). The different *P*_CO_ in each experiment may lead to small inconsistencies in the carbon deposition as well as the time required for achieving steady states (Table S5). Nevertheless, the general picture on the direction of the electron flow via EMSCI is valid.

### CO Oxidation Kinetics at Four Stages of Electron Flow

EMSCI describes the flow of electrons in the sequence of 1) CO; 2) Cu^0^; 3) carbon + Ce^3+^; 4) O_2_. At these four stages, the Cu oxidation states are Cu^2+^, Cu^0^, Cu^+^ and Cu^+^/Cu^2+^, respectively. The CO oxidation kinetics is then studied at these four stages (Figure S24) to understand the influence of surface carbon and Cu species in catalysis.

**Figure 5:**
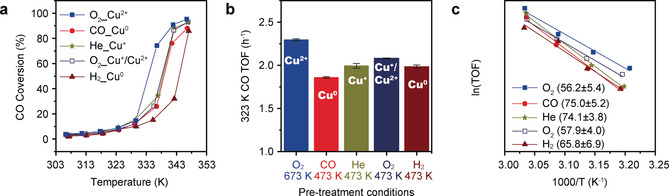
The catalytic performance of 20 wt % CuO‐CeO_2_ in CO oxidation after different pretreatments. CO oxidation conditions: Weight hourly space velocity (WHSV) per gram of catalyst: 1500 mL_CO_ h^−1^⋅g^−1^. 80 mg 20 wt % CuO‐CeO_2_, 200 mL min^−1^ flow, 1 % CO, 10 % O_2_, He balanced. a) Conversion of CO as a function of temperature and catalysed by the catalysts with different Cu oxidation states: Cu^2+^ (Stage 1, blue solid squares); Cu^0^ (Stage 2, red circles); Cu^+^ (Stage 3, dark yellow stars); Cu^+^/Cu^2+^ (Stage 4, navy open squares); H_2_‐reduced Cu^0^ (dark red triangles). b) CO TOF at 323 K. c) Arrhenius plots in the range of 313–328 K. The calculated *E*_a_ values (kJ mol^−1^) are listed in parentheses.

The reaction temperature is controlled below 353 K to preserve the initial states of the catalysts. The distinct CO conversion profiles (Figure 5 a) indicate the different catalytic behaviours at each stage. In general, the CO oxidation activity of the initial Cu species on CeO_2_ follows the order Cu^2+^>Cu^+^>Cu^0^. The fully oxidised CuO‐CeO_2_ shows the highest turnover frequency (TOF) at 323 K and lowest apparent activation energy (*E*_a_) (Figure 5 b,c blue) whereas the Cu^0^‐CeO_2_ shows the lowest activity and increased *E*_a_ (Figure 5 b,c red). The Cu^0^‐CeO_2_ catalysts separately reduced by CO and H_2_ show similar TOF and *E*_a_ in the kinetic region (Figure 5 b,c red and wine). However, the CO‐reduced catalyst with considerable amount of surface carbon shows much higher activity in the high conversion region compared to the H_2_‐reduced catalyst which is free of carbon (Figure 5 a red and wine). It suggests that surface carbon on Cu^0^ species is irrelevant to the activity in the kinetic region but can significantly promote the reaction in the high conversion region. After the CO‐reduced Cu^0^‐CeO_2_ is annealed in He, the obtained Cu^+^ with reduced surface carbon shows increased TOF (Figure 5). The obtained Cu^+^ cannot be fully oxidised to the initial Cu^2+^ in 20 % O_2_/He at 473 K, thus the activity cannot fully recover although its *E*_a_ significantly decreases (Figure 5 a,c navy with open symbol).

The Ce^3+^ content is generally accepted to be inversely proportional to thermodynamic oxygen vacancy formation energy (*E*_vac_)^[23]^ which is regarded as a descriptor for CO oxidation activity.^[32]^ Unfortunately, there is no clear relationship between the Ce^3+^ content and CO oxidation performance can be correlated in this work. It suggests that influence of Ce^3+^ content towards CO oxidation is not as obvious as that of the Cu oxidation state.

In conclusion, the lower oxidation state of Cu species on CeO_2_ leads to inferior CO oxidation activity. Surface carbon is irrelevant to the activity in the kinetic region but can significantly promote the reaction in the high conversion region.

## Conclusion

The concept of EMSCI is established to describe the electron flow for the CuO‐CeO_2_ system during CO oxidation. More specifically, the electrons flow from CO to Cu, then to Ce and surface carbon species, and finally to O_2_. The EMSCI concept sheds light on the catalytic cycle of other CO and CO_2_ involved reactions promoted by polyvalent metal oxides. The probability of transition metals transferring the *d* electrons to the π* orbitals of surface carbon species will govern how CO_2_ or CO can be activated over the catalysts.

## Conflict of interest

The authors declare no conflict of interest.

## Supporting information

As a service to our authors and readers, this journal provides supporting information supplied by the authors. Such materials are peer reviewed and may be re‐organized for online delivery, but are not copy‐edited or typeset. Technical support issues arising from supporting information (other than missing files) should be addressed to the authors.

Supplementary
