# Supplementary material for: The Electrophilicity of Surface Carbon Species in the Redox Reactions of CuO‐CeO2 Catalysts
Source: Angew Chem Int Ed Engl. 2021 Apr 8;60(26):14420–8. doi: 10.1002/anie.202102570 (PMC8251948; doi:10.1002/anie.202102570)
Supplement: Supplementary file 1 — Supplementary [file ANIE-60-14420-s001.pdf]

Angewandte Chemie
Eine Zeitschrift der Gesellschaft Deutscher Chemiker

Supporting Information

The Electrophilicity of Surface Carbon Species in the Redox Reactions of CuO-CeO_2 Catalysts

Liqun Kang[+], Bolun Wang[+], Andreas T. Güntner, Siyuan Xu, Xuhao Wan, Yiyun Liu, Sushila Marlow, Yifei Ren, Diego Gianolio, Chiu C. Tang, Vadim Murzin, Hiroyuki Asakura, Qian He, Shaoliang Guan, Juan J. Velasco-Vélez, Sotiris E. Pratsinis, Yuzheng Guo, and Feng Ryan Wang**

anie_202102570_sm_miscellaneous_information.pdf

Table of Contents

Experimental Procedures 2
Supporting Figures 4
Supporting Tables 18
References 20
Author Contributions 21

Experimental Procedures

Catalysts preparation. The CuO-CeO_2 composites were synthesized with a flame spray pyrolysis (FSP) reactor described elsewhere [1]. The copper and cerium precursor solutions were prepared by mixing appropriate amounts of cerium(III) 2-ethylhexanoate (Sigma-Aldrich, 49wt% in 2-ethylhexanoic acid) with Deca Copper 8 (Borchers GmbH, contains 7.8-8.2 wt% Cu)[2] in a 2:1 (by volume) solution of 2-ethylhexanoic acid (ACROS, 99%) and xylene (Sigma-Aldrich, 95%). The pure CeO_2, 1wt% and 20wt% CuO-CeO_2 catalysts (based on the mass fraction of CuO in the whole composite) were made by tailoring the cerium(III) 2-ethylhexanoate to Deca Copper 8 ratio. The total metal concentration in the precursors was always 0.3 mol·L^{-1}. These precursor solutions were fed through a nozzle at 5 mL·min^{-1} and dispersed by 5 L·min^{-1} O_2 (Pangas, 99,95%) with a pressure drop of 2.0 bar. The resulting fine spray was ignited and sustained by a premixed CH_4/O_2 flamelet (flow rates 1 and 2 L·min^{-1}, respectively) provided through a ring-shaped annulus surrounding the nozzle. The as-prepared flame-made nanoparticles were collected on glass-fibre filters (GF6 Albet-Hahnemuehle, 257 mm diameter) with a vacuum pump at 50 cm height above the burner. Finally, the obtained powders were oxidised in a tubular reactor under a H_2/air mixture flow (5% H_2/N_2 50 mL·min^{-1} diluted by air 150 mL·min^{-1}) at 473 K for 30 minutes.

High-resolution aberration-corrected Annular Bright Field (ABF) and high resolution aberration-corrected high angle annular dark field-scanning transmission electron microscopy (HAADF-STEM) investigations. The data were acquired on probe-corrected (CEOS) JEM ARM 200CF electron microscope (JEOL, Japan) at the electron Physical Science Imaging Centre (ePSIC). The sample was prepared by sprinkling dry catalyst powder on 400-mesh gold grids with lacey carbon film. By using a 20 µm probe-forming aperture, the beam current was 33 pA with a 15.3 mrad probe convergence semi-angle at 200 keV operation voltage. The ABF and HAADF signals were collected simultaneously at 6.0 cm STEM camera length, integrating the scattered electron intensity below 29.3 mrad and between 107.0 to 415.1 mrad, respectively. The regions of interest were exposed to an intense electron beam for 15 min to mitigate the accumulation of carbon contamination during STEM imaging.

Energy-dispersive X-ray spectroscopy (EDS) investigations. The elemental mapping data were acquired in STEM mode on the same microscope by large solid-angle dual EDS detectors. The probe-forming aperture was increased to 40 µm to improve the signal to noise ratio, leading to 143 pA beam current with 31.8 mrad probe convergence semi-angle. The EDS elemental mapping area was 100 × 100 pixels (0.1 s exposure time per pixel) with spatial drifting correction every 30 s. Gatan Microscopy Suite Software was used for EDS spectrum imaging data acquisition.

X-ray photoelectron spectroscopy (XPS) investigations. The data were acquired on a Thermo Fisher Scientific NEXSA spectrometer. The samples were analysed using a micro-focused monochromatic Al X-ray source (72 W) over an area of approximately 400 microns. Data was recorded at pass energies of 200 eV for survey scans, and 50 eV for the high-resolution scan with 1 eV and 0.1 eV step sizes, respectively. Charge neutralization of the sample was achieved using a combination of both low energy electrons and argon ions. The C 1s electron at 284.8 eV was used as a standard reference to calibrate the photoelectron energy shift. XPS spectra in Ce 3d region from 840 eV to 940 eV was collected and fitted to identify the ratio of Ce in different oxidation states. All the data analysis was performed on the CasaXPS software (version: 2.3.18PR1.0).

Near ambient pressure-near edge X-ray absorption fine structure (NAP-NEXAFS) investigations. The Cu L-edge NAP-NEXAFS experiments were accomplished at ISISS beamline of Berliner Elektronenspeicherring-Gesellschaft für Synchrotronstrahlung (BESSY II, Germany) and 8A2 beamline of Pohang Light Source (PLS-II, South Korea). At ISISS beamline, the *in situ* NEXAFS spectra were collected in both Total Electron Yield (TEY) mode and Auger Electron Yield (AEY) mode at different partial pressures. The reaction products were online monitored using an electron impact mass spectrometer (“PRISMA”, PFEIFFER VACUUM GmbH, Asslar (Germany)) connected directly to the main experimental chamber by a leak valve. The pressure in the specimen chamber was precisely controlled (UHV or 0.1-1 mbar) by simultaneous operations of several mass flow controllers for reactive gases and a PID-controlled throttle valve for pumping gas out. Sample pellets (8 mm diameter) were mounted on a sapphire holder and heated uniformly from the back by a focused infrared laser. A stainless-steel plate was placed behind the pellet to improve the heat transfer. The temperature was monitored by a K-type thermocouple and regulated by a PID controller connected to the laser power source. At 8A2 beamline, a similar experiment endstation was used and only NEXAFS spectra in AEY mode were collected by a Scienta SES100 Analyser. The pressure in the specimen chamber was controlled by several leak valves for reactive gases. Sample pellets were mounted on a stainless-steel holder with a built-in thermal couple. Similar heating unit and PID controller was installed as well. Cu L-edge NEXAFS spectra were collected from 920 eV to 970 eV with 0.2 eV step size. The excitation energy scale was calibrated using the absorption edge of metallic Cu (932.67 eV at the adsorption edge inflexion point).

The C K-edge NAP-NEXAFS study was performed at B07-1 beamline of Diamond Light Source (DLS, UK). The 1wt% CuO-CeO_2 sample was prepared by drop-casting of sample powder dispersed in H_2O solution on Au coated Si. NEXAFS spectra were collected from 275 eV to 310 eV with 0.2 eV step size. All the *in situ* NEXAFS experiments were performed with the degassing of sample in UHV for at least 1 hour before any reactive gas was introduced. The drop-casting 1wt% CuO-CeO_2 sample had a significant charging issue. Moreover, the CFF value was changed from 2.0 to 1.4 to inhibit the third-order absorption of Ce, which partially overlaps with carbon absorption. These two factors caused the excitation energy of gas-phase CO to shift from 287.3 eV[3] to 290.8 eV. Therefore, all the spectra obtained at the C K-edge with 1wt% CuO-CeO_2 were calibrated by -3.5 eV.

X-ray absorption fine structure (XAFS) investigations. Cu K-edge (8.979 keV) X-ray absorption near edge structure (XANES) was performed at B18 beamline of Diamond Light Source (DLS, UK).[4] A QEXAFS mode was set-up through a fast-scanning Si (111) double crystal monochromator (DCM) and Pt-coated branch of collimating and focus mirrors. A couple of Pt-coated harmonic rejection mirrors were inserted between the monochromator and ion chamber to cut off the photons with higher energy. The photon flux at 8 keV (near Cu K-edge at 8.797 keV) was 5×10^{11} ph/s with a beam size of 200 (H)×250 (V) µm. The XAFS spectra with an energy range of 8780-9880 keV were collected in transmission mode using ion chamber detectors. Cu foil was used for energy shift calibration. CuO and

Cu_2O standards were diluted with boron nitride and pressed into pellets for transmission measurement. Cu K-edge ***Operando* XANES** of 20wt% $CuO-CeO_2$ catalysts was performed in a plug-flow microreactor. The catalysts powder was diluted with boron nitride and packed into a Kapton foil reaction tube (diameter 6 mm) with quartz wool at both ends. The reaction tube was connected to the gas supply system. 5vol% CO in He, 5vol% O_2 in He and pure He was supplied through three mass flow controllers under ambient pressure. The outlet gases were sampled continuously with the Quadrupole Mass Spectrometer Quantitative Gas Analyser (Hiden Analytical, UK). A K-type thermal couple was inserted into the catalyst bed to monitor the temperature. A hot air gun was placed under the reaction tube to heat the catalyst bed (heating and cooling ramp rate of 10 °C·min^{-1} and 20 °C·min^{-1} respectively). The heating zone was sheathed with an additional ceramic drivepipe to improve the heat conductivity and prevent uneven heating. Two 3 mm × 15 mm windows were placed on both sides of the ceramic drivepipe to let X-rays pass through. The time resolution of each scan was 2.5 min/spectrum (k_{max} = 17, step size 0.3 eV).

Ce L_3-edge (5.724 keV) XANES was performed at the BL01B1 beamline of Super Photon ring - 8 GeV (SPring-8, Japan) and P64 beamline of Deutsches Elektronen Synchrotron (DESY, German). XAFS spectra (5.525 - 6.150 keV) were measured in transmission mode using Si (111) DCM and ion chambers. V and Cr foil were used for energy shift calibration. Ce L_3-edge *Operando* XANES of 20wt% $CuO-CeO_2$ catalysts was performed in a self-made stainless steel plug-flow microreactor provided by BL01B1 beamline or a 0.5mm diameter quartz tube plug-flow microreactor provided by P64 beamline. 5vol% CO in He, 10vol% O_2 in He and pure He was supplied through three mass flow controllers under ambient pressure. The outlet gas compositions were monitored by the mass spectrometers provided by these two beamlines, respectively. The time resolution of each scan was 2.5 min/spectrum (k_{max} = 10, step size 0.3 eV).

XAFS data was analysed using Demeter software package (including Athena and Artemis methods, version 0.9.26). Athena was used for data normalization and XANES peak fitting.

Synchrotron X-ray powder diffraction (SXPD) investigations. The *in situ* study of $CuO-CeO_2$ catalysts for CO oxidation was carried out at Beamline I11 in Diamond Light Source (UK).[5] A home-designed reaction cell with a quartz capillary (diameter of 1.0 mm) with its ends sealed by epoxy to two brass tubes which were connected to Swagelok fittings. Quartz wool was placed in both ends of the capillary to prevent sample movement during gas flow. Gas tubing was connected on both sides of the fittings. The cell assembly was supported by a metal stent to keep the alignment and to prevent the break of the quartz tube. A monochromatic beam of calibrated wavelength = 0.826115 ± 0.000010 Å) from a pattern of Si (SRM640c) standard was used to obtain X-ray diffraction data (from 2θ = 2 – 92 ° with 0.004 ° step size). Each data scan took 2 seconds, and the scan interval was set to 8 seconds. The sequential powder patterns were obtained using the fast Position Sensitive Detector (PSD) [5]. The time interval of each scan is 10s to capture the full redox process in 10 minutes. The sample was studied with a gas mixture of CO (2 vol% in He), pure He and O_2 (2 vol% in He) flowing through the powders. Pure CeO_2 nanoparticles synthesized by the same flame spray method was measured at room temperature and used for background subtraction for the diffraction patterns of 20wt% $CuO-CeO_2$. Laboratory based X-ray diffraction (XRD) measurements were performed on a Bruker D8 diffractometer with a voltage of 40 kV at 30 mA, using a Cu source with $K_{\alpha 1}$ = 1.540562 Å.

Diffuse reflectance infrared Fourier transform spectroscopy (DRIFTS) investigations. The data were collected on an Agilent Carey 680 FTIR spectrometer (Agilent, UK) equipped with a liquid nitrogen cooled MCT detector and a Harrick reaction chamber. The catalyst powder was filled in a sample cup and placed inside the reaction chamber. The IR beam was directed into it by the Praying Mantis accessory. The time resolution of each spectrum is 60 s (400–4,000 cm^{-1}), while 64 scans were taken and merged to improve the signal to noise ratio. The outlet gas was analysed by the same Hiden QGA mass spectrometer.

CO reduction, temperature programmed desorption (TPD) and oxidation (TPO) investigations. The experiments were performed on the same FD-2000 reactor**.** Typically, 80-100 mg $CuO-CeO_2$ was put into a quartz tube. The content of CO and CO_2 in the exhaust gas was quantified by AO2000 analysers. To remove any potential carbon contamination, the sample was pre-oxidised in 100 mL·min^{-1} 5% O_2/He at 673 K for 30 mins. After oxidation, the sample was cooled down to room temperature in He. For CO-TPD, the sample adsorbed CO in 2% CO/N_2 (100 mL·min^{-1}) at room temperature for 30 mins and was purged with He (100 mL·min^{-1}) for another 30 mins to remove physically adsorbed CO. The CO adsorbed sample was then heated to 673 K in He with a rate at 5 K·min^{-1}, then kept at 673 K for 30 mins. For O_2-TPO, the sample was kept in 5% O_2/He flow (100 mL·min^{-1}) and heated from 300 K to 673 K with a rate at 5 K·min^{-1}, then kept at 673 K for 30 mins.

Catalytic tests. The catalytic performance in CO oxidation was evaluated on a FD-2000 fix-bed reactor (Huasi, China). Approximately 80 mg catalyst powder was packed into a quartz tube. A mixture of gases (1vol% CO, 10vol% O_2, balanced by He) was introduced to the reactor via 2 MFCs under the flow of 200 ml·min^{-1}, leading to weight hourly space velocity (WHSV) per gram of catalysts equals to 1,500 mL_{CO}·h^{-1}·g^{-1}. The exhaust gas was analysed by AO2000 Series Advance Optima Continuous Gas Analyser (ABB, Germany) equipped with 3 individual sensors: IR spectrometer for CO and CO_2, superparamagnetic O_2 analyser and thermal conductivity detector for H_2. The catalyst at individual stage (Figure S24) was obtained under certain pre-treatment conditions, then cooling to room temperature. The CO oxidation with these catalysts is tested from room temperature to 353 K.

Computational methods. Spin-polarized density functional theory (DFT) calculations are carried out by Perdew-Burke-Ernzerhof (PBE) functional within generalized gradient approximation (GGA) in the Vienna ab initio Simulation Package (VASP).[6] The projector-augmented-wave (PAW) basis set[7] was adopted to describe ion-electron interaction with a cut-off energy of 520 eV. Only Γ point was used for reciprocal space integration. The convergence tolerance of energy and force was 10^{-5} eV and 0.02 eV/Å, respectively. Grimme's DFT-D3 method was incorporated to include the van der Waals interaction.[8] Bader charge analysis is performed to describe the charge variation quantitatively.[9] To investigate the structure variation, ab initio molecular dynamics (AIMD) simulations are performed from 3000 K to 300 K with NVT ensemble.

SUPPORTING INFORMATION

Supporting Figures

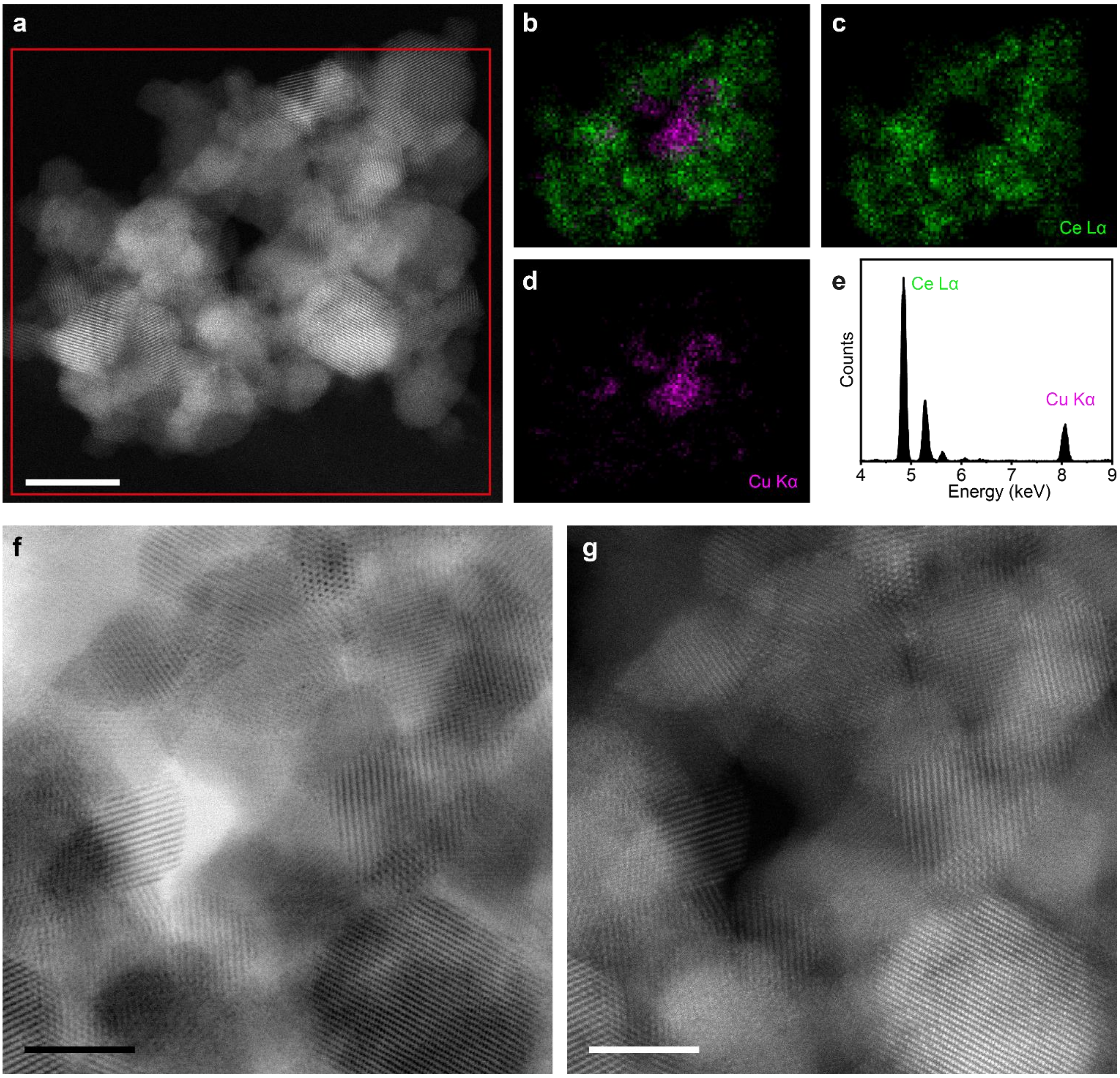

Figure S1. The HAADF-STEM images of pristine 20wt% CuO-CeO_2 (a, f, g) and the EDS mapping (b-e). The pristine catalyst consists of 5.1 nm Cu clusters (average of 50 particles) and 5.8 nm CeO_2 clusters (average of 100 particles). Due to the low Z-contrast of Cu compared with Ce, the blur of Cu fringes may induce errors in measuring the diameter. As the particle sizes of CuO and CeO_2 clusters are less relevant to the electronic metal-support-carbon interactions (EMSCI) proposed in this work, we did not count more particles to enhance the accuracy. Scale bars: 10 nm in (a) and 5 nm in (f, g).

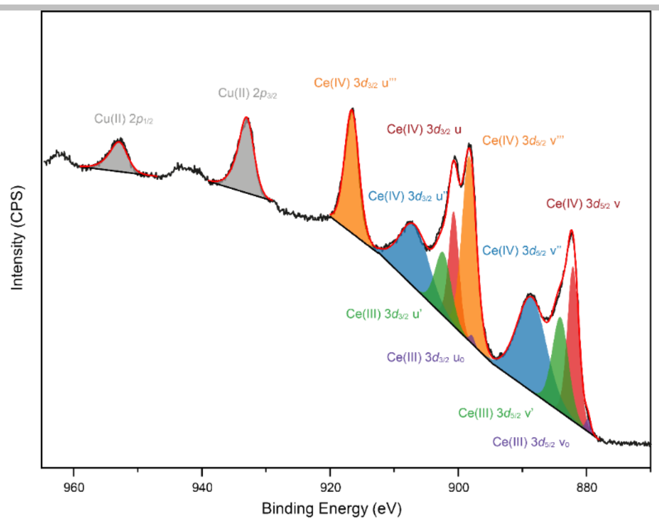

Figure S2. The XPS spectrum of 20wt% CuO-CeO_2 in Cu 2*p* and Ce 3*d* region. The fitting results are listed in Table S1.

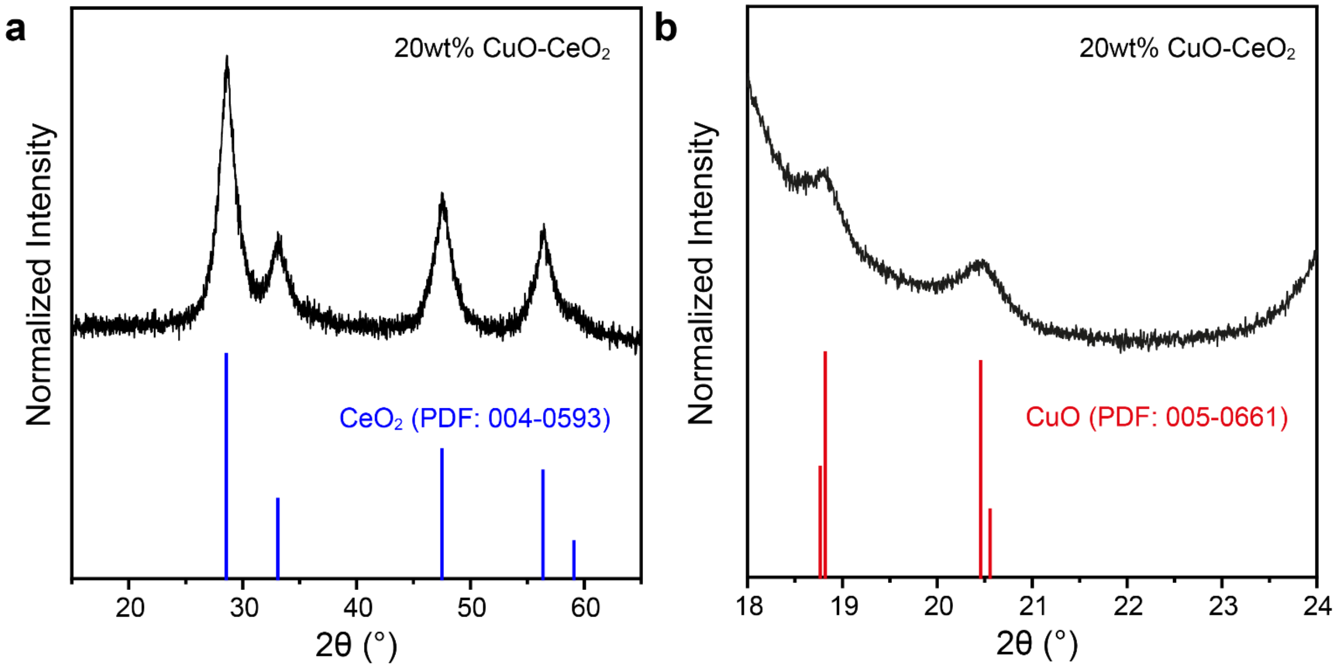

Figure S3. The X-ray diffraction (XRD) and synchrotron X-ray powder diffraction (SXPD) patterns of 20wt% CuO-CeO_2**.** (a) The XRD pattern of 20wt% CuO-CeO_2 measured with Cu Kα radiation (λ = 1.5418 Å). The diffraction peak positions for CeO_2 standard (PDF 004-0593: blue bars) are marked at 28.56° (111), 33.09° (200), 47.50° (220), 56.36° (311) and 59.11° (222). (b) A small portion of the SXPD pattern of 20.0wt% CuO-CeO_2 obtained at I11 beamline of Diamond Light Source (λ = 0.826115 ± 0.000010 Å). The diffraction peak positions for monoclinic CuO standard (PDF 005-0661: red bars) are marked at 18.78° (002), 18.84° ($11\bar{1}$), 20.47° (111) and 20.57° (200).

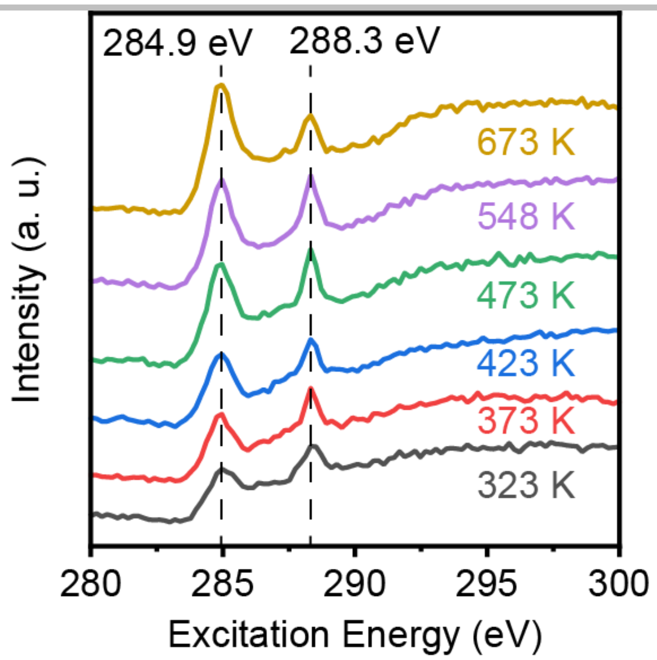

Figure S4. Carbon K-edge NEXAFS of 1wt% $CuO-CeO_2$ in UHV. The ratio of reduced carbon species (284.9 eV) versus oxidised carbon species (288.3 eV) increases with increasing temperature. The 1wt% $CuO-CeO_2$ was synthesised *via* the same FSP method reported in our previous study.[10]

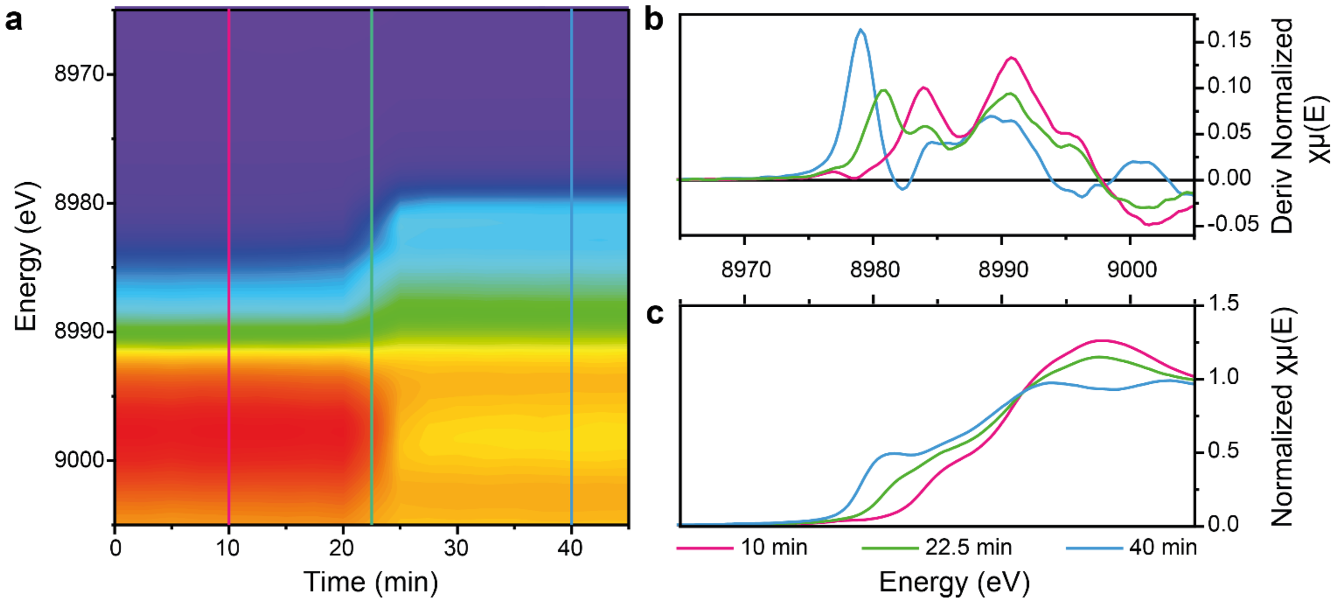

Figure S5. *Operando* Cu K-edge X-ray absorption near edge structure (XANES) in the CO stage at 453 K. (a) Contour map of the Cu K-edge XANES spectra when the atmosphere is switched from O_2 to CO. (b) The first derivative of the XANES spectra of $CuO-CeO_2$ in O_2 (10 min, pink curve), intermediate Cu^+ species (22.5 min, green curve) and Cu^0-CeO_2 in CO (40min, blue curve). (c) The XANES spectra of $CuO-CeO_2$ in O_2 (10 min, pink curve), intermediate Cu^+ species (22.5 min, green curve) and Cu^0-CeO_2 in CO (40min, blue curve).

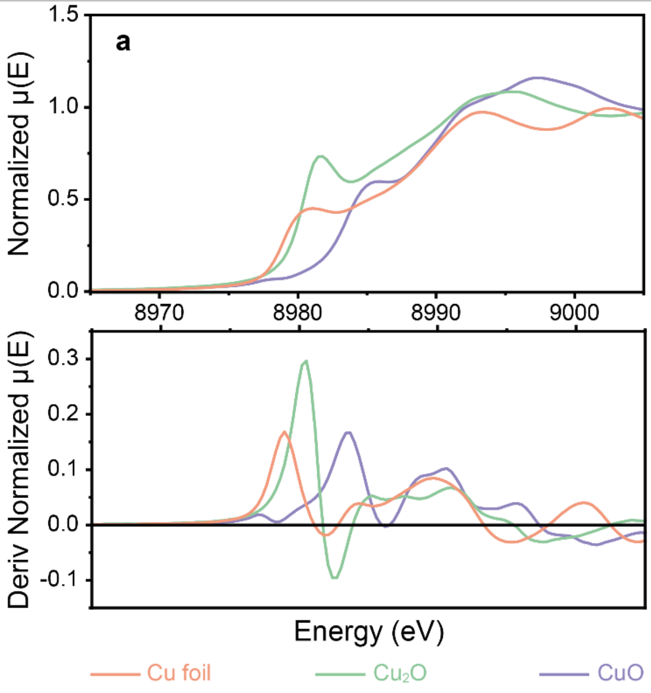

Figure S6. Cu K-edge XANES spectra (a) and the corresponding (b) first derivative of CuO, Cu_2O and Cu foil standards.

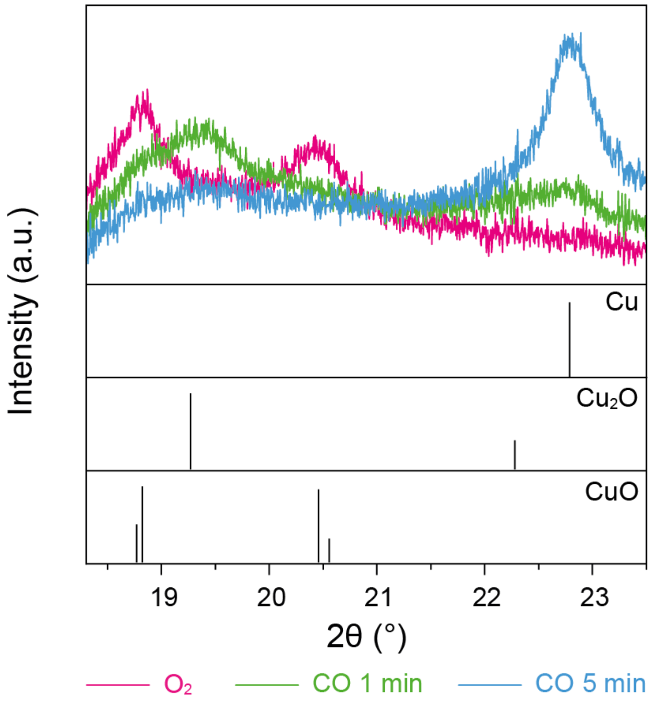

Figure S7. The *in situ* SXPD patterns of initial CuO in O_2, intermediate Cu_2O species and Cu^0-CeO_2 in the CO stage at 453 K. The XRD patterns of standard Cu species are shown below.

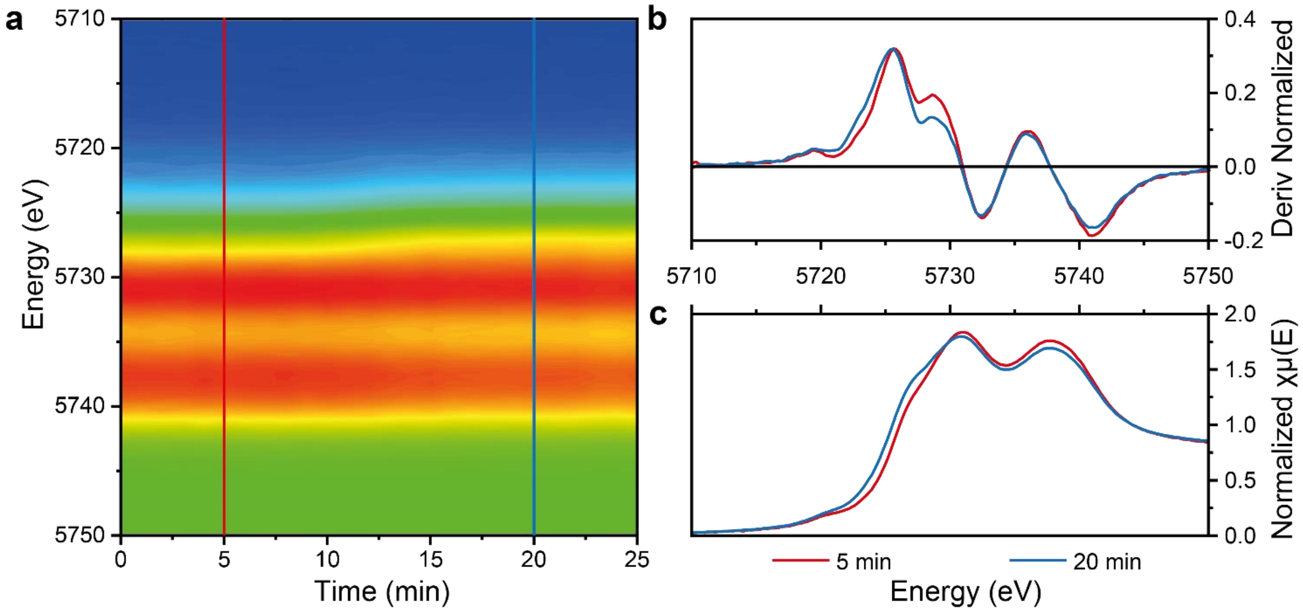

Figure S8. *Operando* Ce L_3 edge XANES in the CO stage at 453 K. (a) Contour map of the Ce L_3 edge XANES spectra when the atmosphere is switched from O_2 to CO. (b) The first derivative of the XANES spectra in O_2 (5 min, red curve) and in CO (20 min, blue curve). (c) The XANES spectra in O_2 (5 min, red curve) and in CO (20 min, blue curve). Their fitting curves are shown in Figure S9A and S9B.

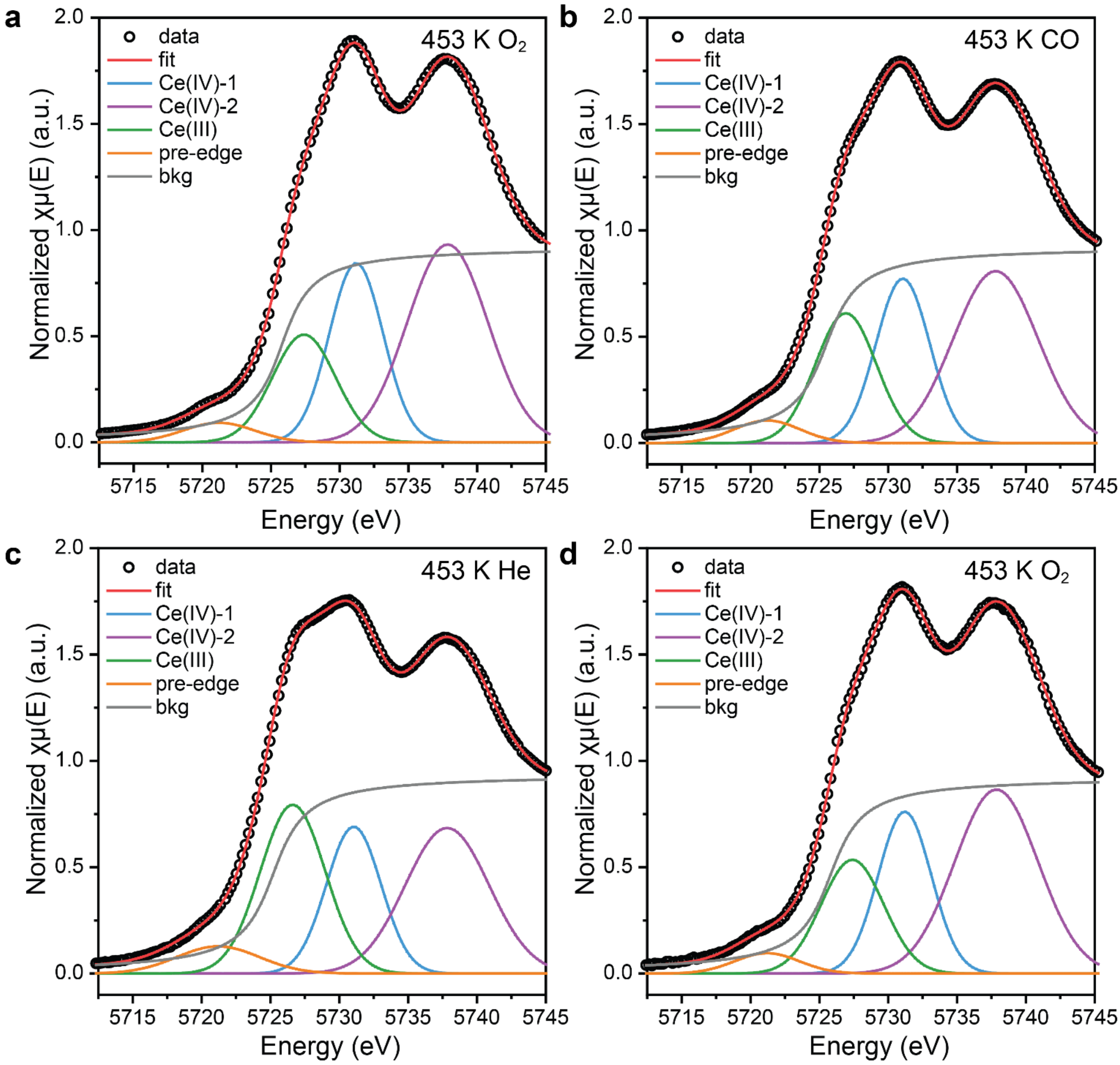

Figure S9. The fitting results of *in-situ* Ce L_3 edge XANES spectra of 20wt% CuO-CeO_2. (a) Initial state in 5% O_2 at 453 K. (b) In 5% CO at 453 K. (c) In He at 453 K. (d) After reoxidation, in 5% O_2 at 453 K. The detailed fitting results are listed in Table S3.

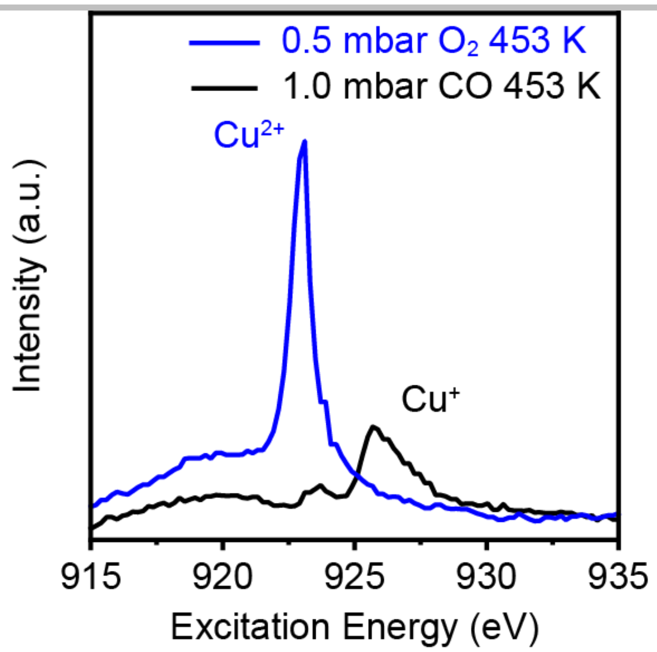

Figure S10. NAP-NEXAFS shows the surface Cu^{+} formed during the reduction of 20wt% CuO-CeO_2 in 1.0 mbar CO at 453 K.

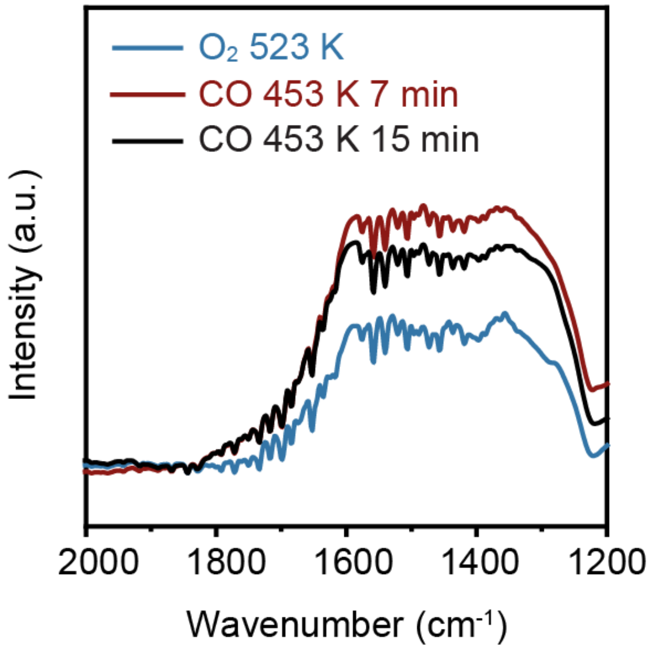

Figure S11. The *in situ* diffuse reflectance infrared Fourier transform spectroscopy (DRIFTS) shows the change of carbonates during the reduction of CuO-CeO_2 in 1% CO/He at 453 K

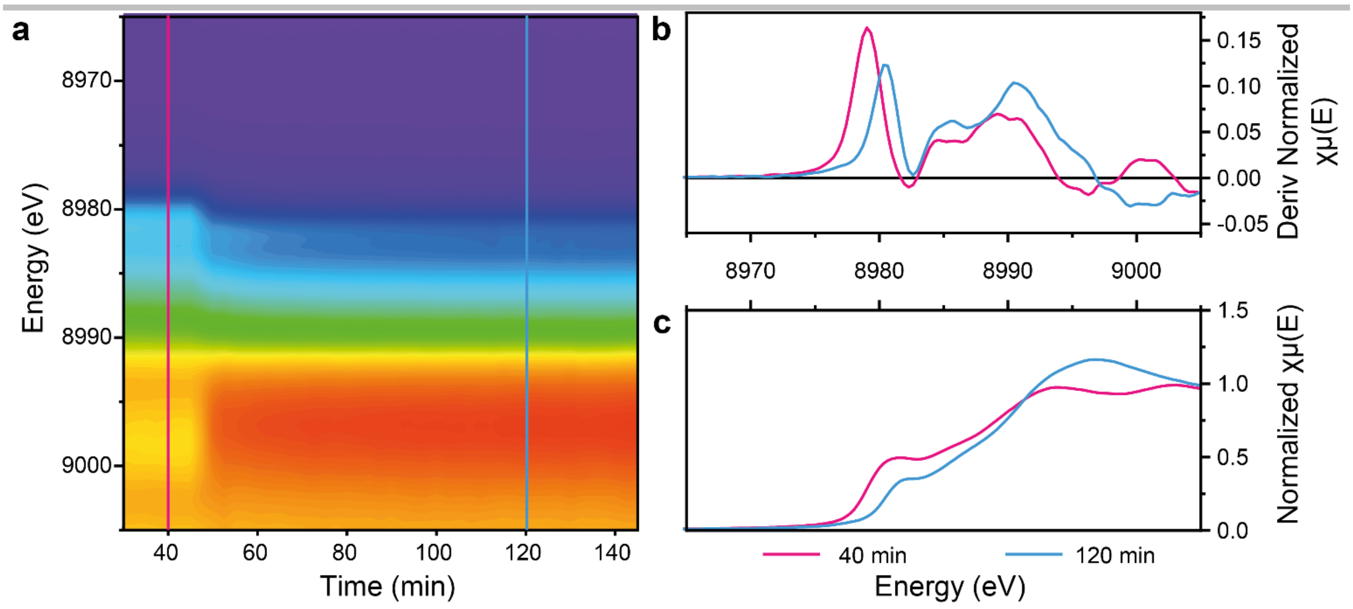

Figure S12. *Operando* Cu K-edge XANES in the He stage at 453 K. (a) Contour map of the Cu K-edge XANES spectra when the atmosphere is switched from CO to He. (b) The first derivative of the XANES spectra of Cu^0-CeO_2 in CO (40 min, pink curve), and Cu^+-CeO_2 in He (120 min, blue curve). (c) The XANES spectra of Cu^0-CeO_2 in CO (40 min, pink curve), and Cu^+-CeO_2 in He (120 min, blue curve).

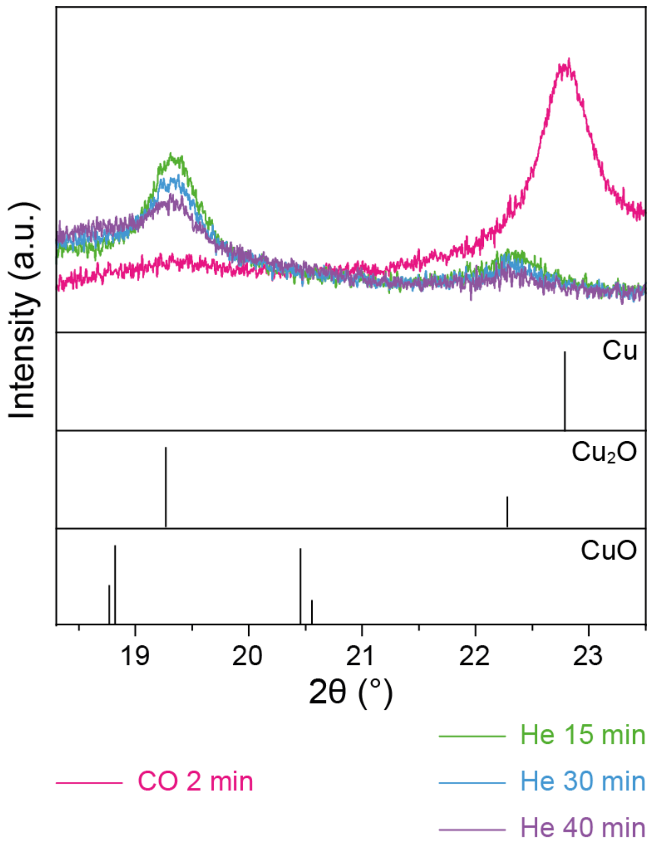

Figure S13. The *in situ* SXPD patterns of the conversion of metallic Cu to crystalline Cu_2O and the amorphization of Cu_2O in He at 453 K.

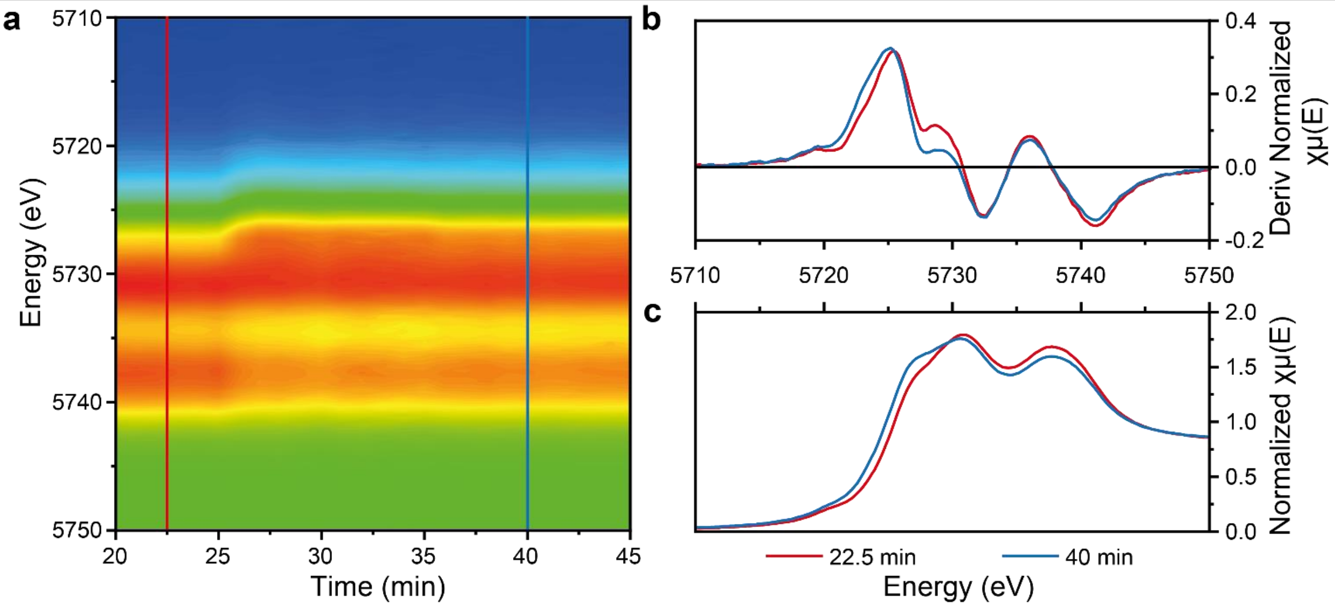

Figure S14. *Operando* Ce L_3 edge XANES in the He stage at 453 K. (a) Contour map of the Ce L_3 edge XANES spectra when the atmosphere is switched from CO to He. (b) The first derivative of the XANES spectra in CO (22.5 min red curve), and in He (40 min, blue curve). (c) The XANES spectra in CO (22.5 min red curve), and in He (40 min, blue curve). Their fitting curves are shown in Figure. S9B and S9C.

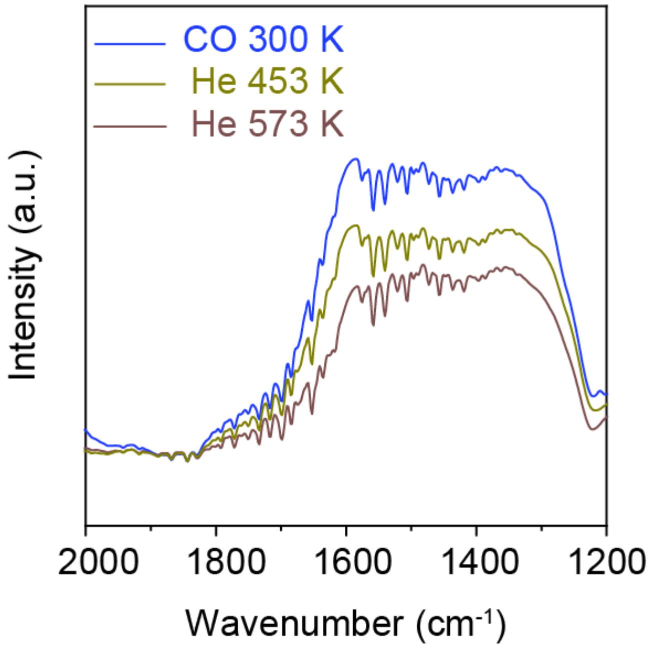

Figure S15. The *in situ* DRIFTS shows the decomposition of surface carbonates from 300 K to 573 K.

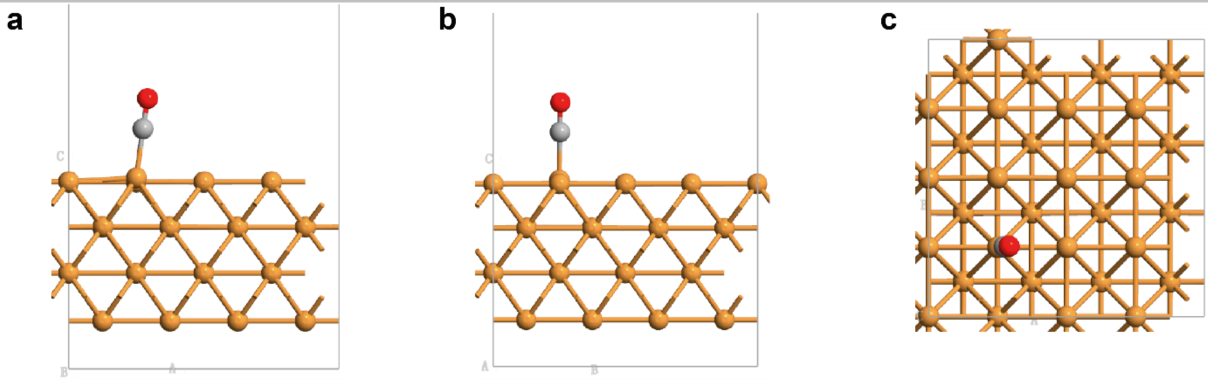

Figure S16. The theoretical calculations of one CO molecule on metallic Cu surface.

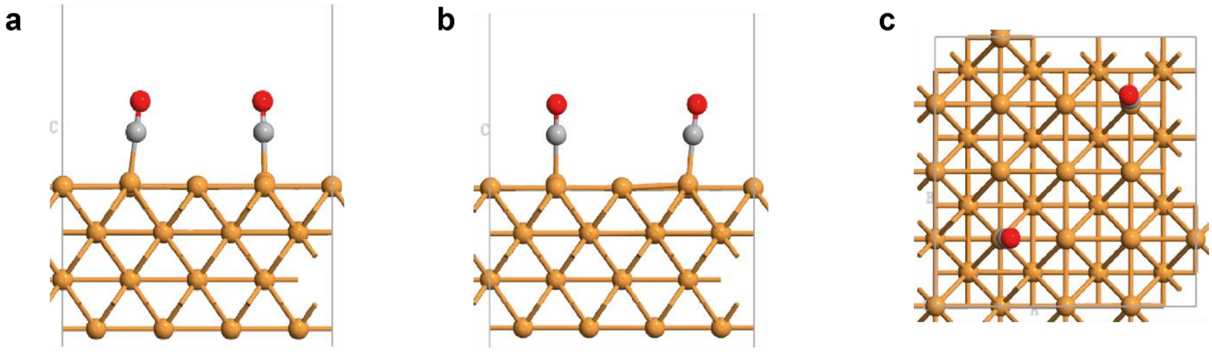

Figure S17. The theoretical calculations of two CO molecules on metallic Cu surface.

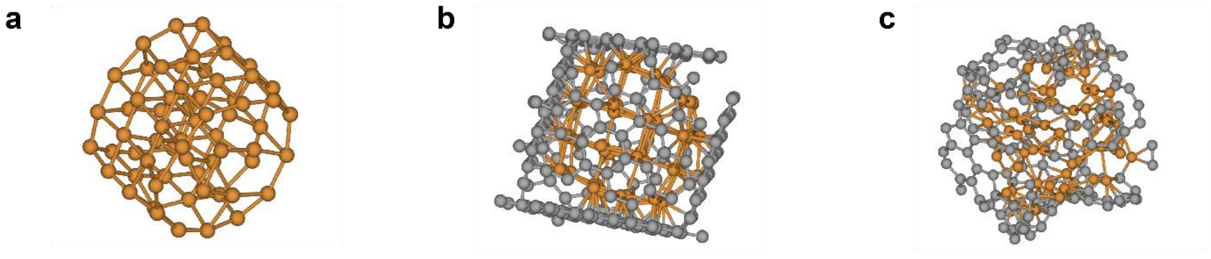

Figure S18. The theoretical calculations of carbon on metallic Cu cluster. (a) Cu cluster; (b) Cu cluster with carbon; (c) Cu cluster with carbon after molecular dynamics simulation.

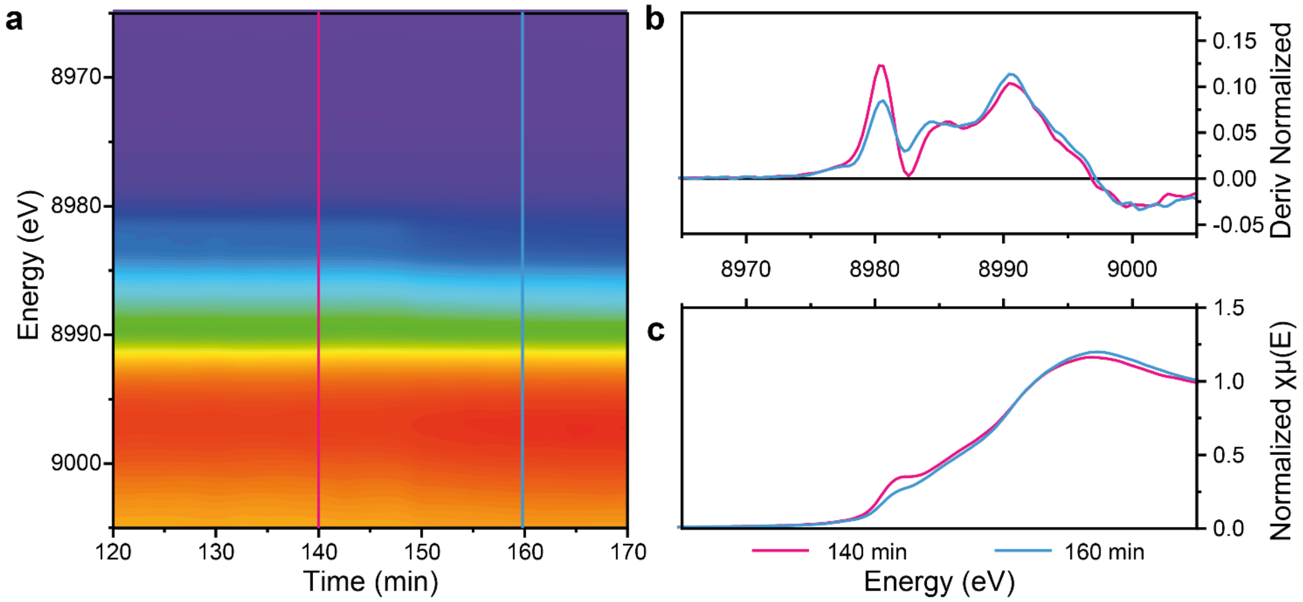

Figure S19. *Operando* Cu K-edge XANES in the O_2 stage at 453 K. (a) Contour map of the Cu K-edge XANES spectra when the atmosphere is switched from He to O_2. (b) The first derivative of the XANES spectra of Cu^+-CeO_2 in He (140 min, pink curve), and Cu^{2+}/Cu^+-CeO_2 in O_2 (160 min, blue curve). (c) The XANES spectra of Cu^+-CeO_2 in He (140 min, pink curve), and Cu^{2+}/Cu^+-CeO_2 in O_2 (160 min, blue curve).

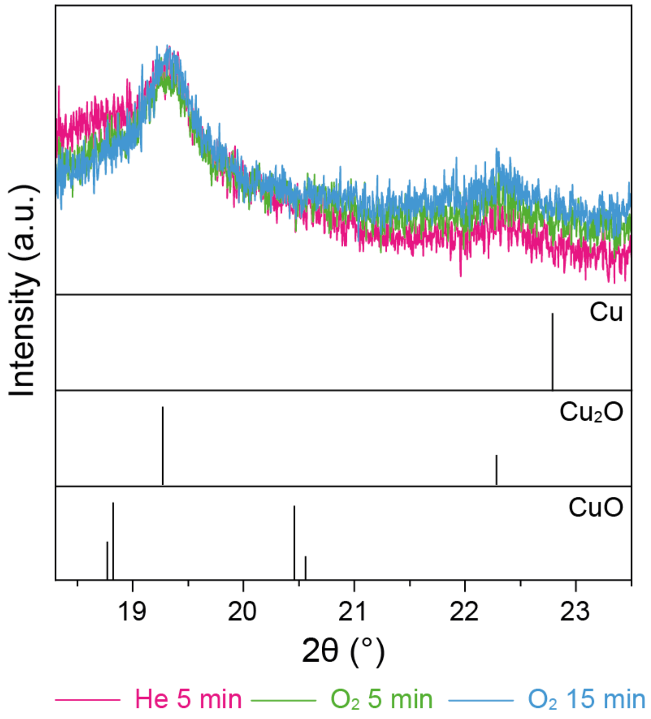

Figure S20. The *operando* SXPD patterns of the preserved crystalline Cu_2O in He and 2% O_2/He at 453 K.

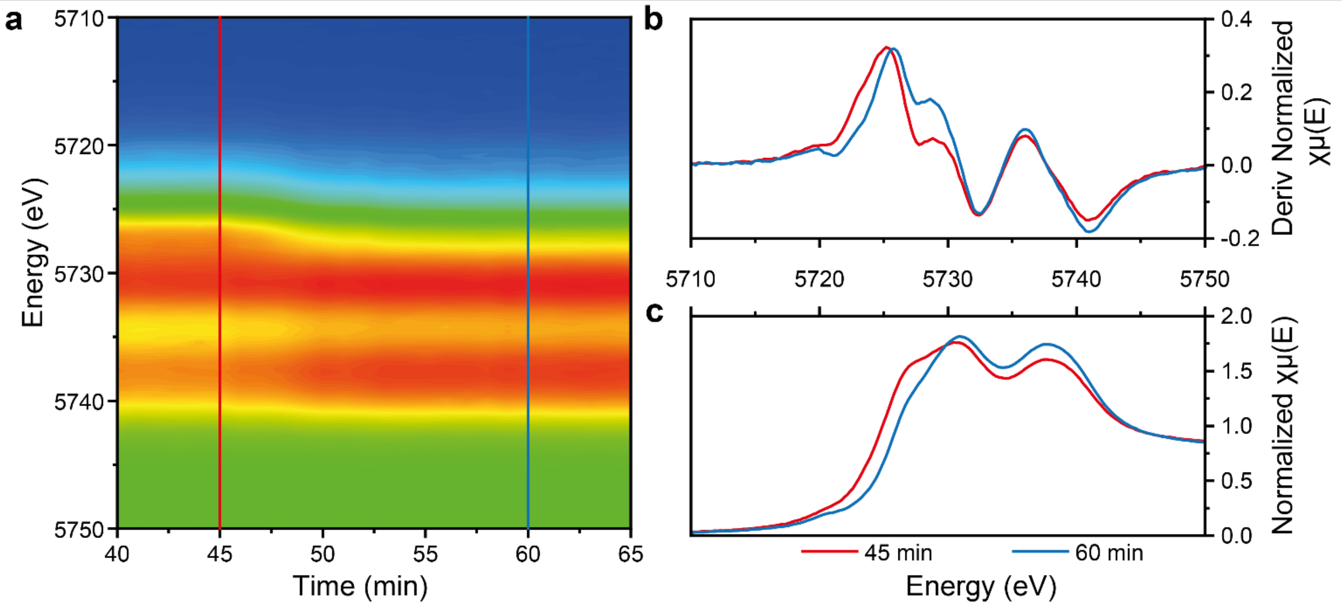

Figure S21. *Operando* Ce L_3 edge XANES in the O_2 stage at 453 K. (a) Contour map of the Ce L_3 edge XANES spectra when the atmosphere is switched from He to O_2. (b) The first derivative of the XANES spectra in He (45 min, red curve) and in O_2 (60 min, blue curve). (c) The XANES spectra in He (45 min, red curve) and in O_2 (60 min, blue curve). Their fitting curves are shown in Figure S9C and S9D.

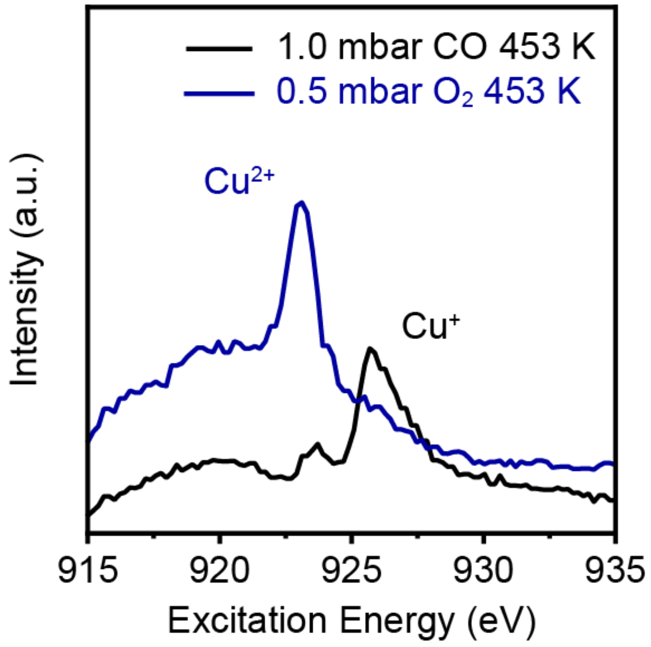

Figure S22. NAP-NEXAFS shows the complete oxidation of surface Cu^{+} to Cu^{2+} at 453 K in 0.5 mbar O_2.

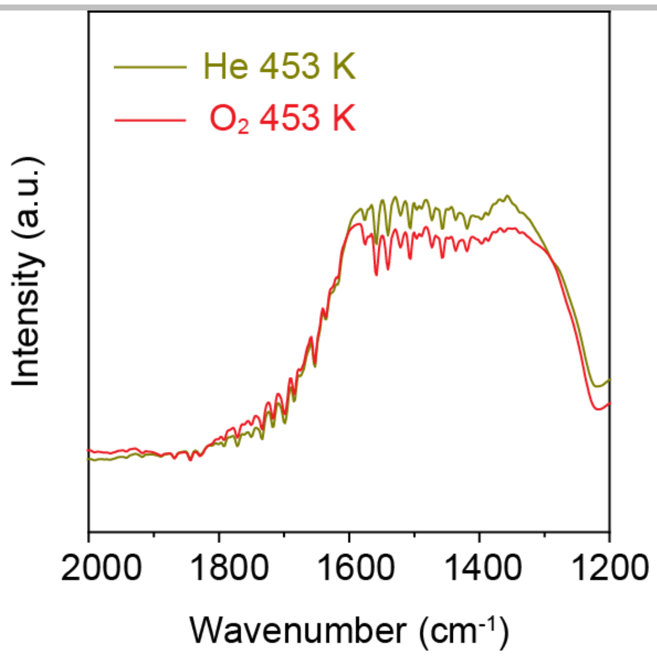

Figure S23. The *in situ* DRIFTS shows the slight decrease of surface carbonates at 453 K in 2% O_2/He.

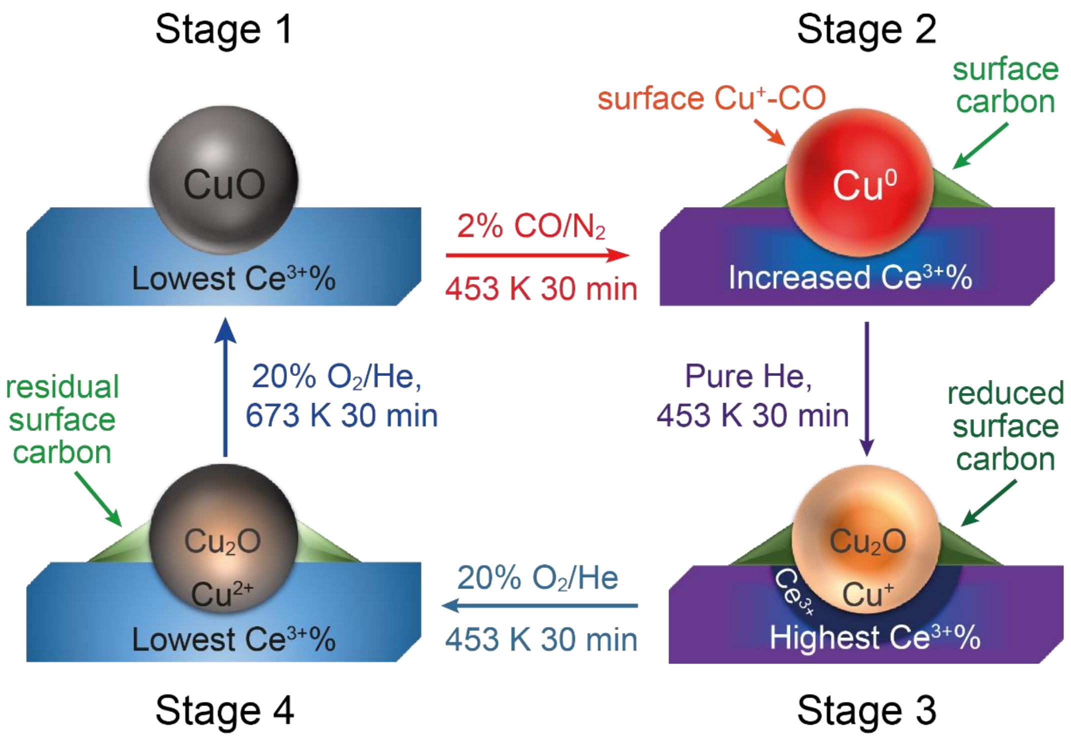

Figure S24. The structural change of the 20% CuO-CeO_2 catalyst after different pre-treatment. The models shown in the schematic are simplified for illustrating the major structural changes.

According to the *in-situ* spectroscopy study, four stages of the catalysts with different Cu oxidation states (Cu^{2+}, Cu^0, Cu^+ and Cu^+/Cu^{2+}) can be obtained after certain pre-treatment (Figure S24).

Cu^{2+} at Stage 1: The 20% CuO-CeO_2 was oxidised in 20% O_2/He at 673 K for 30 minutes to remove any carbon contamination and ensure the Cu species is fully oxidised. Lowest Ce^{3+} content will be obtained under this condition.

Cu^0 at Stage 2: The catalyst from Stage 1 was reduced in 2% CO/N_2 at 453 K for 30 minutes. Metallic Cu can be produced with increased Ce^{3+} content (Figure 2a-c). Surface carbon is deposited from CO in forms of carbonyls and carbonates species (Figure 2d

and Figure S11). To decouple the effects of surface carbon and Cu oxidation states, metallic Cu is also obtained by the reduction in 2% H_2/N_2 at 453 K for 30 minutes, leading to carbon-free Cu^0-CeO_2.

Cu^+ at Stage 3: The catalyst from Stage 2 was annealed in He at 453 K for 30 minutes. Electrons will be transferred from Cu^0 to surface carbon and Ce^{4+}, leading to predominant Cu^+ with maximum Ce^{3+} content and reduced surface carbon (Figure 3).

Cu^+/Cu^{2+} at Stage 4: The catalyst from Stage 3 was oxidised in 20% O_2/He at 453 K for 30 minutes. The initial Cu^{2+} state cannot be fully recovered but the Ce^{3+} content increases to the original level (Figure 4a-c). The surface Cu can be mostly oxidised to Cu^{2+} (Figure S19), whereas the bulk Cu nanoparticle maintains significant feature of Cu_2O (Figure 4a,b). There is still some residual carbon species left on the surface (Figure S20).

Based on these four stages, we have studied the evolution of 20wt% CuO-CeO_2 under different CO/O_2 ratio at 453 K (Figure S25 and Table S6). It is found that the metallic Cu is quickly oxidised to Cu^{2+} with 10-16% Cu^+ when O_2 is excess. Only stage 1 exists under standard CO oxidation condition (1% CO, 10% O_2 and balance He). Therefore, we cannot compare the kinetics of CO oxidation with different Cu oxidation states at high temperature under O_2 rich conditions.

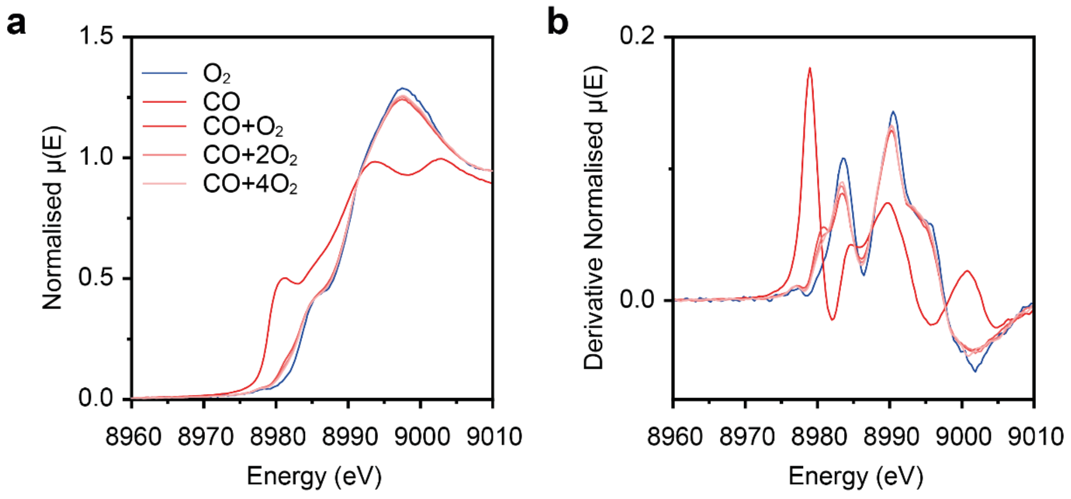

Figure S25. *Operando* Cu K-edge XANES under different atmosphere at 453 K. (a) The XANES spectra. (b) The first derivative of the XANES spectra.

SUPPORTING INFORMATION

Supporting Tables

Table S1. XPS fitting results of 20wt% CuO-CeO_2

Species	Peak	label	Final state	BE [eV]	Area	
Ce (III)	$3d_{5/2}$	v_0	Ce $3d^9 4f^2$ O $2p^5$	879.8	0.5%	18.1%
	$3d_{3/2}$	u_0		897.9	0.3%	
	$3d_{5/2}$	v'	Ce $3d^9 4f^1$ O $2p^6$	884.1	10.4%	
	$3d_{3/2}$	u'		902.4	6.9%	
Ce (IV)	$3d_{5/2}$	v	Ce $3d^9 4f^0$ O $2p^6$	882.1	12.0%	81.9%
	$3d_{3/2}$	u		900.7	8.0%	
	$3d_{5/2}$	v''	Ce $3d^9 4f^1$ O $2p^5$	888.6	19.0%	
	$3d_{3/2}$	u''		906.8	12.7%	
	$3d_{5/2}$	v'''	Ce $3d^9 4f^2$ O $2p^4$	898.3	18.0%	
	$3d_{3/2}$	u'''		916.6	12.1%	
Cu	$2p_{3/2}$	N/A	Cu $2p^5$ O $2p^6$	933.0	66.7%	
	$2p_{1/2}$			952.9	33.3%	

The relative sensitivity factors (R. S. F.) for Ce 3d and Cu 2p are 51.62 and 25.39 (values from CasaXPS), respectively. The peak ratio of Ce(3d) : Cu(2p) = 78.5% : 21.5%, suggesting a surface atomic Cu/(Cu+Ce) ratio of 0.36.

Table S2. The comparison of bulk/surface Cu content in $CuO-CeO_2$ samples prepared by different methods

Preparation method	Cu/(Cu+Ce)	
	Bulk	XPS
Co-precipitation[a]	0.14	0.61
Combustion[a]	0.15	0.55
Citrate-hydrothermal[a]	0.25	0.63
Impregnation[a]	0.15	0.63
FSP in this work	0.35[b]	0.36

[a] Data from literature.[11]

[b] Theoretical value calculated based on the composition of 20wt% $CuO-CeO_2$.

Table S3. Peak fitting results of Ce L_3-edge XANES spectra.

**Sample**	**Peak A**	**Peak B**	**Peak C**	**Peak D**
**Energy**	**5737.8 eV**	**5731.1 eV**	**5726.8 eV**	**5721.2 eV**
453 K, O_2[a]	47.3%	28.5%	20.1%	4.1%
453 K CO	44.4%	26.9%	24.2%	4.5%
453 K He	36.5%	24.0%	32.8%	6.7%
453 K O_2[b]	48.1%	27.4%	20.6%	3.9%

[a] Initial state before the CO reduction.

[b] Final state after the reoxidation.

Peak fitting results of Ce L_3 edge XANES. The peak fitting was performed with an arctangent function to simulate the edge jump and Gaussian function for the absorption of different transition. The centre of the arctangent function was set at the maximum point in first derivative XANES, which is the inflexion point of the main edge. Four peaks have been included for the fitting. Peak A and B at 5737.8 and 5731.1 eV were associated with Ce(IV), corresponding to final states of $4f^0 5d^1$ and $4f^1 5d^1$ L, respectively (L refers to a ligand charge transfer to 5d orbitals). Peak C can be referred to Ce(III) absorption, and Peak D in the pre-edge region is due to the dipole-forbidden $2p_{3/2}$ to 4f transition, which is a result of a 5d orbital mixture with the 4f state. The ratio of Ce(III) and Ce(IV) in the samples were calculated using the following equations:
Ratio of Ce(III) = Peak C /(Peak A + Peak B + Peak C + Peak D)

Table S4. The Bader charge analysis of metallic Cu atoms and adsorbed carbon species.

Model	Cu atom with CO	The rest Cu atoms	Each CO molecule	C atoms
Cu + one CO	-0.11e	-0.02e	+0.13e	\
Cu + two CO	-0.12e	-0.01e	+0.13e	\
Cu + carbon	\	-16.58e	\	+16.58e

Table S5. The differences in the time sale of the in-situ spectra.

Major Structural Change	Cu^{2+} to Cu^0	Cu^0 to Cu^+	Cu^+ to Cu^+/Cu^{2+}	Time resolution[b]
Time scale for Cu K-edge XANES[a]	6 min	3 min	9 min	3 min
Time scale for SXPD[a]	4 min	6 min	NA	1 min
Time scale for Ce L_3 edge XANES[a]	6 min	3 min	9 min	3 min
Time scale for DRIFTS[a]	15 min	10 min	8 min	1 min

[a] Time needed for reaching steady state.

[b] Minimum time needed for one spectroscopy.

Due to the limit of synchrotron beamline setup, we cannot obtain XANES, SXPD and DRIFTS at the same time in one beamline. The sample loading amount, gas flow conditions (dead volume, partial pressure and pressure drop) may vary based on the different *operando* cells. Therefore, the P_{CO} of each experiment cannot be controlled to be exactly the same. These inconsistencies result in the differences in the time scale for the reduction from Cu^{2+} to Cu^0 (Figure 2a,b).

In addition, the dominant structural changes shown in XANES, SXPD and DRIFTS may not happen simultaneously. When Cu^0 is converted into Cu^+, the crystalline Cu^0 firstly turns into amorphous Cu^+ which is observed in XANES after 3 minutes (Figure 3a). The feature of crystalline Cu_2O in SXPD appears after 6 minutes (Figure 3b) because the crystallization of Cu_2O does not happen simultaneously with the formation of Cu^+. After being heated in He for 10 minutes, the weakened SXPD feature indicates the amorphization of Cu_2O while the XANES feature of Cu^+ does not change (Figure 3a,b). Meanwhile, the absorption peak of surface carbon reaches steady state in DRIFTS (2127 cm^{-1}) after being heated in He for 10 minutes (Figure 3d), suggesting the decomposition of surface carbon is also not simultaneous with the formation of Cu^+. When Cu^+ is partially oxidised to Cu^{2+}, the XAENS feature of Cu^+ decreases in 9 minutes whereas the feature of crystalline Cu_2O remains unchanged (Figure 4a,b), suggesting mainly amorphous Cu^+ is oxidised.

The electron flow in the catalysts during CO oxidation, especially when CO and O_2 co-exist in the system, may be different from the reduction/oxidation sequence in this study. The kinetics of the redox process of Cu, Ce and surface carbon may be changed by the competitive adsorption of CO and O_2. Nevertheless, since surface carbon species are inevitably formed during the conversion of carbonaceous reactants, the surprising electrophilicity of surface carbon species demonstrated in this work undoubtedly opens new perspectives on the role of coke in catalysis. In addition to Cu-based catalysts, computational simulations suggest that the electronic perturbations from surface carbon species is a general phenomenon for a wide range of transition metals,[12] which make up the majority of active sites for C1 chemistry. The electronic interaction between these transition metal sites and *in situ* formed carbon species can lower the metal *d*-band centre, weakening the adsorption strength of CO[13] and C=C,[14] thus leading to enhanced activity of CO oxidation[15] and improved selectivity towards alkene in alkyne hydrogenation,[16] respectively. Therefore, the positive role of the "notorious" coke in catalysis should be recognised. These *in situ* formed carbon moieties near to the active sites can be regarded as a secondary support[17] and should be optimised based on EMSCI.

Table S6. The content of Cu^0, Cu^+, Cu^{2+} under different atmosphere at 453 K determined by linear combination fitting of Cu K-edge XANES.

Atmosphere	Cu^0 (%)	Cu^+ (%)	Cu^{2+} (%)
O_2	0	0	100
CO	100	0	0
CO+O_2	0	16.1	83.9
CO+2O_2	0	12.4	87.6
CO+4O_2	0	10.4	89.6

References

[1] L. Mädler, W. J. Stark, S. E. Pratsinis, *J. Mater. Res.* **2002**, *17*, 1356-1362.
[2] O. Waser, M. Hess, A. Güntner, P. Novák, S. E. Pratsinis, *J Power Sources* **2013**, *241*, 415-422.
[3] P. Parent, C. Laffon, F. Bournel, J. Lasne, S. Lacombe, *J. Phys.: Conf. Ser.* **2011**, *261*, 012008.

[4] a) A. J. Dent, G. Cibin, S. Ramos, S. A. Parry, D. Gianolio, A. D. Smith, S. M. Scott, L. Varandas, S. Patel, M. R. Pearson, L. Hudson, N. A. Krumpa, A. S. Marsch, P. E. Robbins, *J. Phys.: Conf. Ser.* **2013**, *430*, 012023-012029; b) A. J. Dent, G. Cibin, S. Ramos, A. D. Smith, S. M. Scott, L. Varandas, M. R. Pearson, N. A. Krumpa, C. P. Jones, P. E. Robbins, *J. Phys.: Conf. Ser.* **2009**, *190*, 012039-012042.
[5] S. P. Thompson, J. E. Parker, J. Marchal, J. Potter, A. Birt, F. Yuan, R. D. Fearn, A. R. Lennie, S. R. Street, C. C. Tang, *J. Synchrotron Radiat.* **2011**, *18*, 637-648.
[6] a) G. Kresse, J. Furthmüller, *Phys. Rev. B* **1996**, *54*, 11169-11186; b) J. P. Perdew, K. Burke, M. Ernzerhof, *Phys. Rev. Lett.* **1996**, *77*, 3865-3868.
[7] P. E. Blöchl, *Phys. Rev. B* **1994**, *50*, 17953-17979.
[8] S. Grimme, *Journal of Computational Chemistry* **2006**, *27*, 1787-1799.
[9] W. Tang, E. Sanville, G. Henkelman, *Journal of Physics: Condensed Matter* **2009**, *21*, 084204.
[10] L. Kang, B. Wang, Q. Bing, M. Zalibera, R. Büchel, R. Xu, Q. Wang, Y. Liu, D. Gianolio, C. C. Tang, E. K. Gibson, M. Danaie, C. Allen, K. Wu, S. Marlow, L.-d. Sun, Q. He, S. Guan, A. Savitsky, J. J. Velasco-Vélez, J. Callison, C. W. M. Kay, S. E. Pratsinis, W. Lubitz, J.-y. Liu, F. R. Wang, *Nat. Commun.* **2020**, *11*, 4008.
[11] G. Avgouropoulos, T. Ioannides, H. Matralis, *Appl. Catal. B: Environ.* **2005**, *56*, 87-93.
[12] a) O. Piqué, I. Z. Koleva, F. Viñes, H. A. Aleksandrov, G. N. Vayssilov, F. Illas, *Angew. Chem. Int. Ed.* **2019**, *58*, 1744-1748; b) B. Martínez, O. Piqué, H. Prats, F. Viñes, F. Illas, *Appl. Surf. Sci.* **2020**, *513*, 145765.
[13] I. V. Yudanov, K. M. Neyman, N. Rösch, *Phys. Chem. Chem. Phys.* **2004**, *6*, 116-123.
[14] B. Yang, R. Burch, C. Hardacre, G. Headdock, P. Hu, *J. Catal.* **2013**, *305*, 264-276.
[15] L. Zhang, H. Liu, X. Huang, X. Sun, Z. Jiang, R. Schlögl, D. Su, *Angew. Chem. Int. Ed.* **2015**, *54*, 15823-15826.
[16] a) D. Teschner, J. Borsodi, A. Wootsch, Z. Révay, M. Hävecker, A. Knop-Gericke, S. D. Jackson, R. Schlögl, *Science* **2008**, *320*, 86-89; b) D. Teschner, E. Vass, M. Hävecker, S. Zafeiratos, P. Schnörch, H. Sauer, A. Knop-Gericke, R. Schlögl, M. Chamam, A. Wootsch, A. S. Canning, J. J. Gamman, S. D. Jackson, J. McGregor, L. F. Gladden, *J. Catal.* **2006**, *242*, 26-37.
[17] a) A. Bruix, J. A. Rodriguez, P. J. Ramírez, S. D. Senanayake, J. Evans, J. B. Park, D. Stacchiola, P. Liu, J. Hrbek, F. Illas, *J. Am. Chem. Soc.* **2012**, *134*, 8968-8974; b) J. Graciani, K. Mudiyanselage, F. Xu, A. E. Baber, J. Evans, S. D. Senanayake, D. J. Stacchiola, P. Liu, J. Hrbek, J. Fernandez Sanz, J. A. Rodriguez, *Science* **2014**, *345*, 546-550; c) M. Yoo, Y.-S. Yu, H. Ha, S. Lee, J.-S. Choi, S. Oh, E. Kang, H. Choi, H. An, K.-S. Lee, J. Y. Park, R. Celestre, M. A. Marcus, K. Nowrouzi, D. Taube, D. A. Shapiro, W. Jung, C. Kim, H. Y. Kim, *Energy Environ. Sci.* **2020**, *13*, 1231-1239.

Author Contributions

L.K., B.W. and F.R.W. conceived the study. B.W., A.G. and S.E.P. carried out materials synthesis. L.K. and B.W. performed materials characterization. B.W. and Y. R. performed catalytic evaluation. L.K., B.W., D.G., V.M., H.A., and F.R.W. performed *ex-situ* and *operando* XAS study. L.K., B.W., S.M., Y.L., J.V.V., B.J. and F.R.W. conducted NAP-NEXAFS study. L.K. and Q.H. conducted STEM measurement. S.G. conducted XPS measurement. L.K., C.C.T. and F.R.W measured the SXPD. S. X., X. W. and Y. G. performed theoretical calculations. L.K., B.W. and F.R.W. wrote the manuscript and all authors revised the manuscript.
